# Circulating inflammatory monocytes oppose microglia and contribute to cone cell death in retinitis pigmentosa

**DOI:** 10.1093/pnasnexus/pgac003

**Published:** 2022-03-02

**Authors:** Jun Funatsu, Yusuke Murakami, Shotaro Shimokawa, Shunji Nakatake, Kohta Fujiwara, Ayako Okita, Masatoshi Fukushima, Kensuke Shibata, Noriko Yoshida, Yoshito Koyanagi, Masato Akiyama, Shoji Notomi, Shintaro Nakao, Toshio Hisatomi, Atsunobu Takeda, Eleftherios I Paschalis, Demetrios G Vavvas, Yasuhiro Ikeda, Koh-Hei Sonoda

**Affiliations:** Department of Ophthalmology, Graduate School of Medical Science, Kyushu University, Fukuoka 812-8582, Japan; Department of Ophthalmology, Graduate School of Medical Science, Kyushu University, Fukuoka 812-8582, Japan; Department of Ophthalmology, Graduate School of Medical Science, Kyushu University, Fukuoka 812-8582, Japan; Department of Ophthalmology, Graduate School of Medical Science, Kyushu University, Fukuoka 812-8582, Japan; Department of Ophthalmology, Graduate School of Medical Science, Kyushu University, Fukuoka 812-8582, Japan; Department of Ophthalmology, Graduate School of Medical Science, Kyushu University, Fukuoka 812-8582, Japan; Department of Ophthalmology, Graduate School of Medical Science, Kyushu University, Fukuoka 812-8582, Japan; Department of Ophthalmology, Graduate School of Medical Science, Kyushu University, Fukuoka 812-8582, Japan; Department of Genomics and Molecular Analysis, Yamaguchi University School of Medicine, Yamaguchi 755-8505, Japan; Department of Ophthalmology, Graduate School of Medical Science, Kyushu University, Fukuoka 812-8582, Japan; Clinical Research Center, Saga University Hospital, Saga 849-8501, Japan; Department of Ophthalmology, Graduate School of Medical Science, Kyushu University, Fukuoka 812-8582, Japan; Department of Ophthalmology, Graduate School of Medical Science, Kyushu University, Fukuoka 812-8582, Japan; Department of Ocular Pathology and Imaging Science, Graduate School of Medical Science, Kyushu University, Fukuoka 812-8582, Japan; Department of Ophthalmology, Graduate School of Medical Science, Kyushu University, Fukuoka 812-8582, Japan; Department of Ophthalmology, Graduate School of Medical Science, Kyushu University, Fukuoka 812-8582, Japan; Department of Ophthalmology, Chikushi Hospital, Fukuoka University, Fukuoka 812-8582, Japan; Department of Ophthalmology, Graduate School of Medical Science, Kyushu University, Fukuoka 812-8582, Japan; Department of Ophthalmology, Massachusetts Eye and Ear Infirmary, Harvard Medical School, Boston, MA 02114, USA; Boston Keratoprosthesis Laboratory, Schepens Eye Research Institute, Massachusetts Eye and Ear Infirmary,Harvard Medical School, Boston, MA 02114, USA; Disruptive Technology Laboratory, Department of Ophthalmology, Massachusetts Eye and Ear Infirmary,Harvard Medical School, Boston, MA 02114, USA; Department of Ophthalmology, Massachusetts Eye and Ear Infirmary, Harvard Medical School, Boston, MA 02114, USA; Angiogenesis Laboratory, Department of Ophthalmology, MassachusettsEye and Ear Infirmary,Harvard Medical School, Boston, MA 02114, USA; Department of Ophthalmology, Graduate School of Medical Science, Kyushu University, Fukuoka 812-8582, Japan; Department of Ophthalmology, Faculty of Medicine, University of Miyazaki, Miyazaki 889-1692, Japan; Department of Ophthalmology, Graduate School of Medical Science, Kyushu University, Fukuoka 812-8582, Japan

**Keywords:** neuroinflammation, peripheral monocyte, nanomedicine

## Abstract

Retinitis pigmentosa (RP) is an intractable inherited disease that primarily affects the rods through gene mutations followed by secondary cone degeneration. This cone-related dysfunction can lead to impairment of daily life activities, and ultimately blindness in patients with RP. Paradoxically, microglial neuroinflammation contributes to both protection against and progression of RP, but it is unclear which population(s)— tissue-resident microglia and/or peripheral monocyte-derived macrophages (mφ)— are implicated in the progression of the disease. Here, we show that circulating blood inflammatory monocytes (IMo) are key effector cells that mediate cone cell death in RP. Attenuation of IMo and peripherally engrafted mφ by *Ccl2* deficiency or immune modulation via intravenous nanoparticle treatment suppressed cone cell death in rd10 mice, an animal model of RP. In contrast, the depletion of resident microglia by a colony-stimulating factor 1 receptor inhibitor exacerbated cone cell death in the same model. In human patients with RP, IMo was increased and correlated with disease progression. These results suggest that peripheral IMo is a potential target to delay cone cell death and prevent blindness in RP.

Significance Statement Microglial neuroinflammation has been implicated in retinitis pigmentosa (RP) in both protection against and progression of the disease; however, the immune populations(s) responsible for the progression of RP remain to be elucidated. Here, we show that circulating blood inflammatory monocytes (IMo) oppose tissue-resident microglia and mediate cone cell death in a mouse model of RP. Attenuation of IMo and peripherally engrafted macrophages protects cone cells against degeneration in this model. Blood IMo are increased and associated with the disease progression in patients with RP. Because the loss of cone-mediated daylight vision is the most debilitating aspect of the disease, the results may help in the development of novel immunotherapy to prevent blindness in RP.

## Introduction

Retinitis pigmentosa (RP) is a group of inherited retinal disorders that cause progressive rod and cone degeneration. More than 90 causal genes — most of them exclusively related to rod function, structure, and survival —have been identified as causative for typical RP (i.e. rod–cone dystrophy) (1). The clinical course of the disease initially involves impairment of rod-mediated night vision, followed by the more clinically relevant loss of peripheral and central vision due to secondary cone cell death. Although genetic mutations trigger RP and largely contribute to the pathogenesis of the disease, microenvironmental changes associated with retinal degeneration such as oxidation (2, 3), inflammation (4, 5), and metabolic alterations (6, 7) modulate the disease progression. Gene therapy is emerging as a potential treatment for RP (8, 9), but its application is currently limited by the inability of viral vectors to accommodate large genes and by the progressive degeneration, which occurs even after gene delivery. Moreover, gene correction for dominant mutations is still challenging, and most patients are diagnosed with moderate-to-end stage disease, when most rods are already lost (10, 11). Thus, understanding the biological mechanisms that regulate the disease, and especially the cone cell death, could help improve medical management of RP.

Neuroinflammation is widely associated with and contributes to various forms of neurodegeneration, including RP (3, 12–15). Microglia, a resident macrophage population within the central nervous system (CNS) that is derived from yolk sac progenitors, are the most prominent myeloid cells in the brain and the retina (16). Peripheral blood monocytes do not migrate into the CNS across the healthy blood–brain/retinal barrier, but they can infiltrate and differentiate into macrophages (mφ) in diseased or elderly individuals (3, 14, 15, 17, 18). These resident microglia and peripherally derived mφ are major players in neuroinflammation, and their depletion or inactivation substantially suppresses retinal degeneration in rd10 mice, an animal model of RP (5, 19–21). In contrast, other reports suggest that phagocytic activity of microglia/mφ is critical for the clearance of dying or dead cells and is protective (22–24). These findings suggest that myeloid cells have a dual function to protect against or promote photoreceptor cell death in RP. However, it is still unknown which immune cells or populations have what type of effector function in RP progression, and knowledge about the role of circulating blood inflammatory monocytes (IMo) in RP also remains insufficient.

To dissect the roles of microglia and monocyte-derived mφ in RP, we here investigated the effect of each cell population using a relevant animal model. Moreover, we assessed the levels of IMo in RP patients. Our findings demonstrate that patients and mice with RP exhibit elevated IMo levels in their blood circulation. Attenuation of these cells substantially reduced cone degeneration but not rod degeneration in rd10 mice, an animal model of RP. In contrast, the depletion of resident microglia in the retina by a colony-stimulating factor 1 receptor (CSFR1) inhibitor exacerbates cone cell loss in the same animal model. These results suggest that peripheral IMo are the principal effector cells that mediate cone cell death; whereas resident microglia have an opposing function of protecting cone cells. Thus, inhibiting IMo may be a potential therapy to delay disease progression and prevent blindness in patients with RP.

## Results

### Increased IMo in the peripheral blood of rd10 mice and RP patients

To investigate whether RP is associated with an increase in the number of circulating monocytes, we analyzed the number of CD11b^+^Ly-6C^hi^ IMo in the blood of rd10 mice, a clinically relevant model of RP with *Pde6b* mutation (25, 26). In rd10 mice, rod photoreceptor cell death starts around postnatal day 18 (P18) and most rod cells disappear by P30; this is followed by gradual cone degeneration (25). In the present study, we analyzed the peripheral blood of wildtype (WT) and rd10 mice by flow cytometry at P21, P31, and P42.

**Fig. 1.**
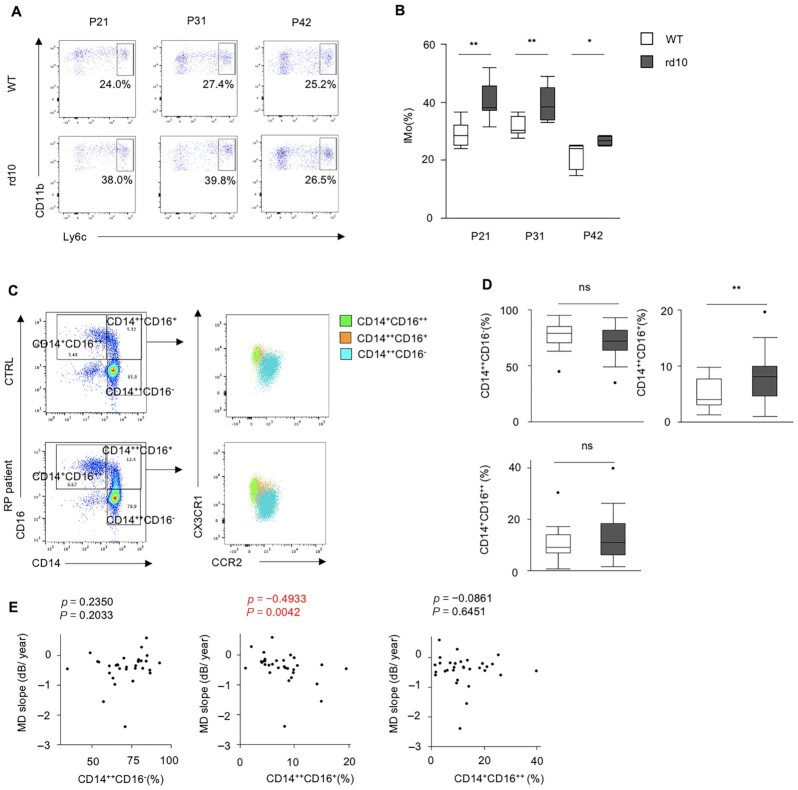
IMo were significantly increased in the peripheral blood of mice and RP patients. (**A**) Flow cytometry findings for the blood samples of WT and rd10 mice at P21, P31, and P42. IMo were defined as CD11b^+^Ly-6C^hi^Ly-6G^lo−neg^ cells. (**B**) The proportions of IMo in WT and rd10 mice (P21: WT, *n* = 7, rd10, *n* = 7; P31: WT, *n* = 8, rd10, *n* = 6; and P42: WT, *n* = 5, rd10, *n* = 5). (**C**) Flow cytometry findings for the blood samples of RP patients (*n* = 31) and healthy controls (*n* = 16). The monocytes were divided into nonclassical (CD14^+^CD16^++^, left upper gate), intermediate (CD14^++^CD16^+^, right upper gate), and classical (CD14^++^CD16^−^, right bottom gate) subsets. The expression levels of CCR2 and CX3CR1 in each subset are shown in the *right panel* (nonclassical subset: *light blue*; intermediate subset: *orange*; and classical subset: *green*). (**D**) The percentages of monocyte subtypes according to CD14/CD16 expression in the healthy subjects and RP patients. CD14^++^CD16^+^ intermediate monocytes were significantly increased in the RP patients compared with the controls. (**E**) The correlations between the mean deviation (MD) slope in HFA10-2 tests and the percentage of monocyte subsets in RP patients were analyzed by Spearman's rank correlation test. There was a negative correlation between the intermediate monocyte subset and the MD slope (i.e. the decline of visual sensitivity). In these graphs, the central horizontal bars indicate the medians, boxes indicate 25th–75th percentiles, and whiskers indicate 1.5 times the interquartile range from the bottom and the top of the box. Outliers are shown as dots. Wilcoxon rank-sum tests were performed to assess the significance. *P < 0.05, **P < 0.01.

The results demonstrated a significant increase in the number of CD11b^+^Ly-6C^hi^Ly-6G^lo^ IMo in rd10 mice at P21 (*P* < 0.01), P31 (*P* < 0.01), and P42 (*P* < 0.05) compared with that of the WT mice (Figure 1A and B). A significant portion of the Ly-6C^hi^ cells also expressed high levels of CCR2 and CX3CR1 markers (Figure S1A, Supplementary Material).

We next evaluated monocyte changes in the RP patients. The subjects' peripheral blood samples were analyzed by flow cytometry, and monocytes were classified into CD14^+^CD16^++^ nonclassical, CD14^++^CD16^+^ intermediate, and CD14^++^CD16^−^ classical monocytes (27). The results revealed no significant difference in the proportion of total monocytes between the RP patients and controls, but subset analysis showed a significant increase in the percentage of CD14^++^CD16^+^ intermediate monocytes in RP patients (*P* = 0.0098, Figure 1C and D; Figure S1B, Supplementary Material). This subset of monocytes co-expressed high levels of CCR2 and CX3CR1 (Figure S1A, Supplementary Material), and high level co-expression of these two chemokine receptors was also observed in mouse Ly-6C^hi^ IMo. Taken together, these findings suggest that RP is associated with an increase in peripheral blood IMo in both humans and animal carriers of the disease.

Subsequent analysis demonstrated a negative correlation between the percentage of intermediate monocytes and mean deviation (MD) slope in the static perimetry tests (i.e. the rate of decline of retinal sensitivity in the macular area; Figure 1E), suggesting that CD14^++^CD16^+^ intermediate monocytes may be related to cone degeneration in RP.

### Changes of microglia and mφ in rd10 mice

To investigate the recruitment of IMo into the retina of rd10 mice, we performed a flow cytometry analysis of the retinal myeloid cells, as described by O'Koren et al., which showed that retinal microglia and mφ can be distinguished by using the surface markers CD45, CD11c, F4/80, and I-A/I-E (28). Accordingly, we stained retinal cells with CD11b, CD11c, CD45, Ly6C, Ly6G, CCR2, and CX3CR1.

**Fig. 2.**
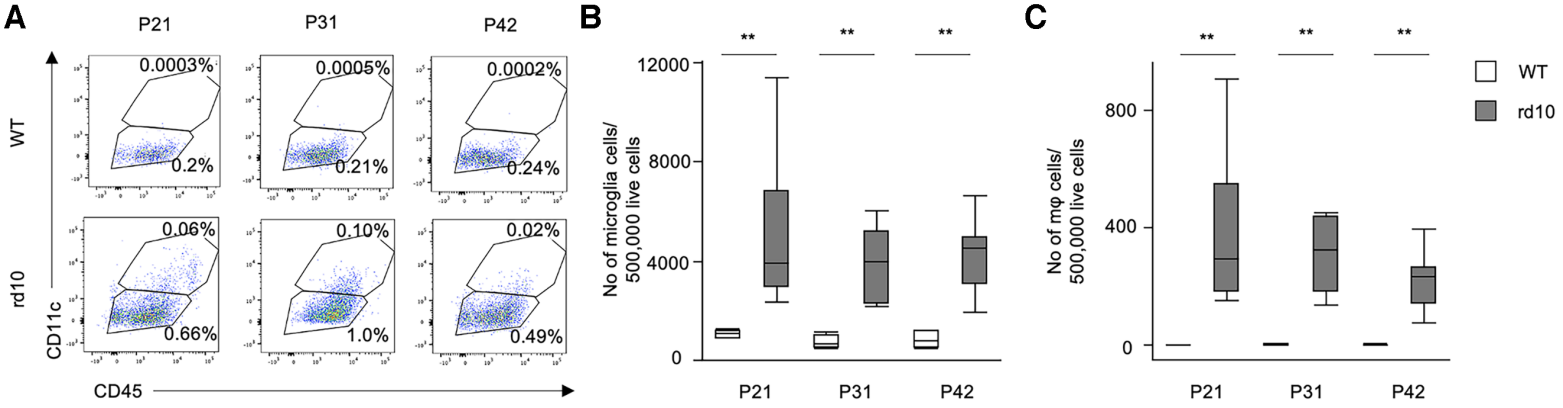
Retinal microglia and mφ were significantly increased in rd10 mice. (**A**) Flow cytometry analysis of the retinal samples of WT and rd10 mice at P21, P31, and P42. CD11b^hi^CD11c^mid^CD45^mid^Ly-6G^lo^Ly-6C^lo^ cells were defined as microglia (lower gate), and CD11b^hi^CD11c^hi^CD45^hi^Ly-6G^lo^Ly-6C^lo^ cells were defined as mφ (upper gate). (**B**) The number of retinal microgila cells per 500,000 analyzed cells of the WT and rd10 mouse retina (P21: WT, *n* = 8, rd10, *n* = 7; P31: WT, *n* = 9, rd10, *n* = 6; and P42: WT, *n* = 7, rd10, *n* = 9). (**C**) The number of mφ cells per 500,000 analyzed cells of the WT and rd10 mouse retina (P21: WT, *n* = 8, rd10, *n* = 7; P31: WT, *n* = 9, rd10, *n* = 6; and P42: WT, *n* = 7, rd10, *n* = 9). The central horizontal bars indicate the medians, boxes indicate 25th–75th percentiles, and whiskers indicate 1.5 times the interquartile range from the bottom and the top of the box. Wilcoxon rank-sum tests were performed to assess the significance. **P < 0.01.

In the WT mice, CD11b^hi^CD11c^mid^CD45^mid^Ly-6G^lo^Ly-6C^lo^ microglia, but not CD11b^hi^CD11c^hi^CD45^hi^Ly-6G^lo^Ly-6C^lo^ mφ, were present in the retina. In contrast, the rd10 mouse retinas had increased numbers of both cell populations at P21, P31, and P42 (all *P* < 0.01; Figure 2A–C). The detailed information including the numbers/percentages of microglia and mφ is shown in Table S1 (Supplementary Material). In line with a previous study (28), the expression levels of F4/80 and MHC class II (I-A/I-E) were higher in peripherally engrafted mφ compared with resident microglia in rd10 mouse retina (Figure S2E, Supplementary Material).

### The CCL2/CCR2 axis mediates IMo and mφ recruitment and contributes to cone cell death in rd10 mice

**Fig. 3.**
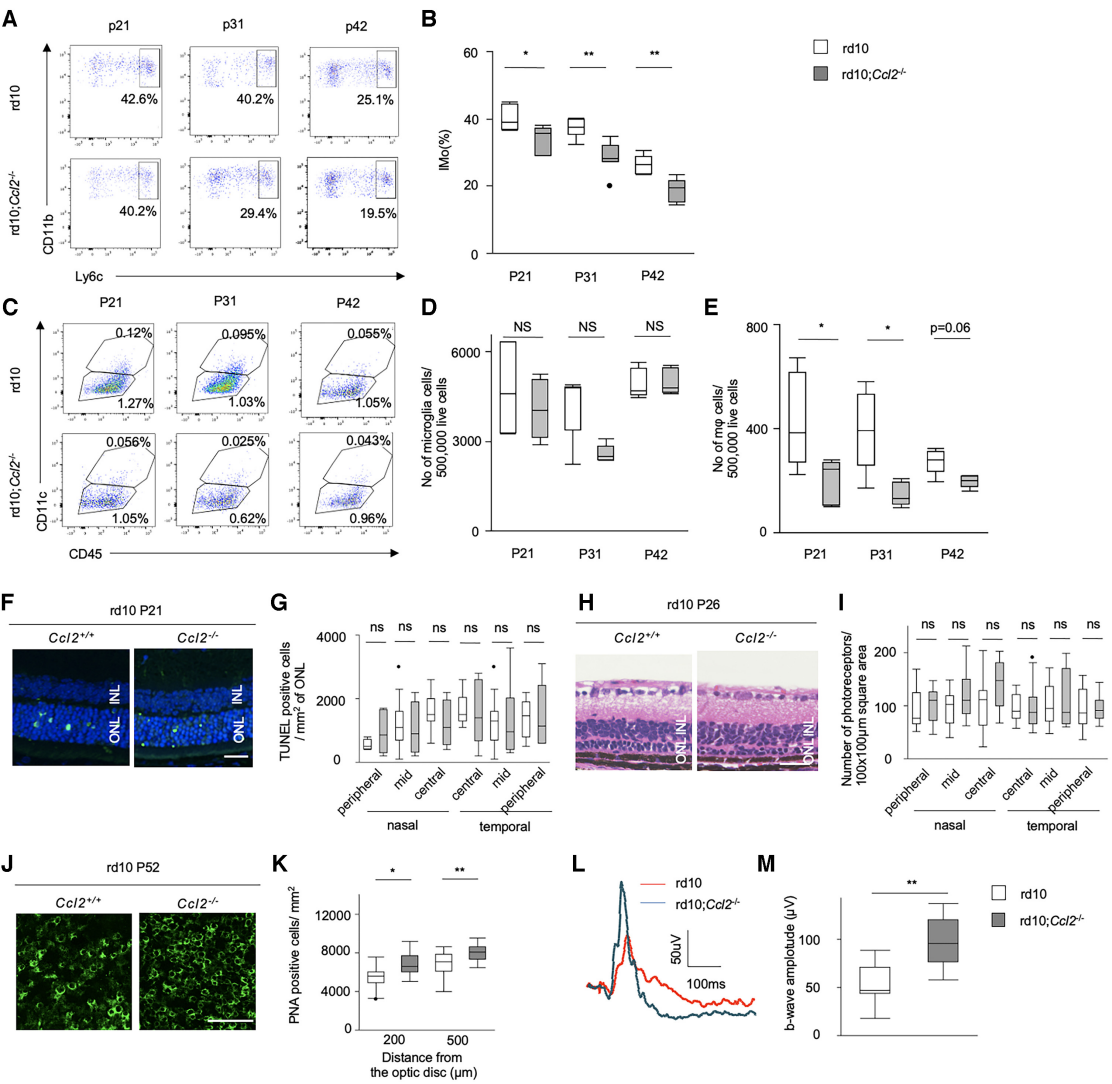
*Ccl2* deficiency-attenuated IMo/mφ recruitment and suppressed cone cell death in rd10 mice. (**A**) Flow cytometry analysis of the blood samples of rd10 and rd10; *Ccl2*^−/−^ mice at P21, P31, and P42. (**B**) The proportions of CD11b^+^Ly-6C^hi^Ly-6G^lo−neg^ IMo of rd10 mice and rd10; *Ccl2*^−/−^ mice (P21: rd10, *n* = 6, rd10; *Ccl2*^−/−^, *n* = 5; P31: rd10, *n* = 7, rd10; *Ccl2*^−/−^, *n* = 7; and P42: rd10, *n* = 5, rd10; *Ccl2*^−/−^, *n* = 7). (**C**) Flow cytometry analysis of the retinal samples of rd10 mice and rd10; *Ccl2*^−/−^ mice at P21, P31m, and P42. The cells surrounded by the lower gate were defined as microglia, and the cells surrounded by the upper gate were defined as mφ. (**D**) The difference in the number of retinal microgila cells per 500,000 analyzed cells between rd10 mice and rd10; *Ccl2*^−/−^ mice at P21: rd10, *n* = 6, rd10; *Ccl2*^−/−^, *n* = 6; at P31: rd10, *n* = 5, rd10; *Ccl2*^−/−^, *n* = 5; and at P42: rd10, *n* = 5, rd10; *Ccl2*^−/−^, *n* = 5. (**E**) The difference in the number of retinal mφ cells per 500,000 analyzed cells between rd10 mice and rd10; *Ccl2*^−/−^ mice at P21: rd10, *n* = 6, rd10; *Ccl2*^−/−^, *n* = 6; at P31: rd10, *n* = 5, rd10; *Ccl2*^−/−^, *n* = 5; and at P42: rd10, *n* = 5, rd10; *Ccl2*^−/−^, *n* = 5. (**F**) TUNEL staining (*green*) and (**G**) quantification of TUNEL-positive photoreceptor cells in the retina of P21 rd10 mice (*n* = 8) and rd10; *Ccl2*^−/−^ mice (*n* = 8). Scale bar: 50 μm. (**H**) Histological findings of the retina and (**I**) the results of the quantitative analysis of photoreceptor cells in the retina of P26 rd10 mice (*n* = 20) and rd10; *Ccl2*^−/−^ mice (*n* = 10). Scale bar: 50 μm. (**J**) PNA staining and (**K**) the quantification of PNA-positive cone photoreceptor cells in the retina of P42 rd10 mice (*n* = 21) and rd10; *Ccl2*^−/−^ mice (*n* = 15). Scale bar: 50 μm. (**L**) Photopic ERG and (**M**) the quantification of b-wave amplitudes at P35 of rd10 mice (*n* = 6) and rd10; *Ccl2*^−/−^ mice (*n* = 8). In these graphs, the central horizontal bars indicate the medians, boxes indicate 25th–75th percentiles, and whiskers indicate 1.5 times the interquartile range from the bottom and the top of the box. Outliers are shown as dots. Wilcoxon rank-sum tests were performed to assess the significance. *P < 0.05, **P < 0.01.

To investigate the roles of IMo and peripherally derived mφ, we blocked the CCL2/CCR2 axis, which is essential for recruiting bone marrow cells into the blood and diseased foci (29), and we created rd10 mice that were deficient for *Ccl2* (rd10; *Ccl2*^−/−^). In the peripheral blood of the rd10 mice, *Ccl2* deficiency decreased the population of IMo at P21, P31, and P42 (*P* < 0.05, *P* < 0.01, and *P* < 0.01, respectively; Figure 3A and B). In the retinas of rd10; *Ccl2*^−/−^ mice, mφ were significantly decreased at P21 (*P* < 0.05) and at P31 (*P* < 0.05), and marginally decreased at P42 (*P* = 0.06). On the other hand, the proportions of resident microglia were not significantly different between the rd10; *Ccl2*^−/−^ and rd10; *Ccl2*^+/+^ mice (Figure 3C–E). Whole-mount immunostaining for Iba-1 showed that Iba-1-positive microglia/macrophages were observed in both inner- and outer-to-subretinal layers of the rd10; *Ccl2*^+/+^ mouse retina (Figure S3A and B, Supplementary Material). In contrast, Iba-1-positive cells were decreased more significantly in the inner retinal layer of rd10; *Ccl2*^−/−^ mice (Figure S3A and B, Supplementary Material). A previous study using fate mapping in a light injury model showed that resident microglia in the retina migrate to the subretinal space; while peripherally engrafted mφ infiltrate into the inner retina during retinal degeneration (18). Taken together, these data show that the CCL2/CCR2 axis is important for IMo/mφ recruitment in rd10 mice.

We next evaluated the effect of *Ccl2* deficiency on rod and cone degeneration in rd10 mice. TUNEL staining at P21, when the rod cell death peaked, showed no significant difference in the number of TUNEL-positive cells in the outer nuclear layer (ONL) between rd10; *Ccl2*^−/−^ and rd10; *Ccl2*^+/+^ mice (Figure 3F and G). Consistent with this finding, the HE staining at P26 demonstrated that there was no significant difference in ONL thickness between rd10; *Ccl2*^−/−^ and rd10; *Ccl2*^+/+^ mice (Figure 3H and I), suggesting that *Ccl2* deficiency may not influence rod degeneration in rd10 mice. In contrast, the cone cell density, as assessed by peanut agglutinin (PNA) labeling, was significantly higher in the P52 rd10; *Ccl2*^−/−^ mice compared with the rd10; *Ccl2*^+/+^ mice (Figure 3J and K). In addition, we analyzed cone function by photopic electroretinogram (ERG). The photopic ERG b-wave was significantly maintained in rd10; *Ccl2*^−/−^ mice compared with rd10 mice (Figure 3L and M). These results suggest that the CCL2/CCR2 axis facilitates recruitment and engraftment of peripheral mφ into the retina, which in turn contributes to cone degeneration in rd10 mice.

### Neuroinflammatory gene profile associated with IMo/mφ attenuation in the rd10 mouse retina

To characterize the neuroinflammatory changes associated with IMo/mφ attenuation, we performed retinal mRNA profiling in WT, rd10; *Ccl2*^+/+^, and rd10; *Ccl2*^−/−^ mice by using a NanoString Neuroinflammation panel containing 757 genes (Figure S4A, Supplementary Material). There were 355 differentially expressed genes (DEGs; *P* < 0.05) between the WT and rd10; *Ccl2*^+/+^ mice, and known inflammatory molecules associated with neurodegeneration, e.g. *Apoe*, *Tnfa*, and *C4a*, as well as chemokines/receptors were upregulated in the rd10 mouse retina (Figure S4B and Table S2, Supplementary Material) (30, 31). Gene set enrichment analysis (GSEA) demonstrated that rd10 mouse retina enriched the gene sets related with interferon-α response, complement, and interferon-γ response (a family-wise error rate < 0.05; Figure S4C, Supplementary Material).

In addition, 84 DEGs between the rd10; *Ccl2*^+/+^ and rd10; *Ccl2*^−/−^ mice were detected (Figure S4D, Supplementary Material). There were 58 DEGs shared in common between these 355 and 84 DEGs (Figure S4E, Supplementary Material). The heat map in Figure S4F (Supplementary Material) shows the top 10 DEGs that were significantly changed in the rd10 vs. the WT retina and reversed by *Ccl2* deficiency.

Several of these 10 most DEGs play important roles in neuroinflammation. *Snca* (synuclein-α) is known to accumulate in Lewy bodies in Parkinson's disease and to promote microglia/mφ activation in neurodegeneration (32). *Tcirg*1 (T cell immune regulator 1) is highly expressed in myeloid cells, including microglia/mφ, and it mediates their activation (33). *Egr1* (early growth response 1) and *Nfkb1* (nuclear factor-κb subunit 1) induce the transcription of pro-inflammatory cytokines, and EGR1 activation promotes neuroinflammation and degeneration in experimental Parkinson's disease (34). *Bnip3* (BCL2 interacting protein 3) is a mitochondrial pro-death molecule that induces autophagy and produces pro-inflammatory cytokines (35).

These results suggest that the attenuation of IMo/mφ by *Ccl*2 deficiency alters the retinal neuroinflammatory status in rd10 mice.

### Microglia depletion exacerbates cone cell loss in rd10 mice

**Fig. 4.**
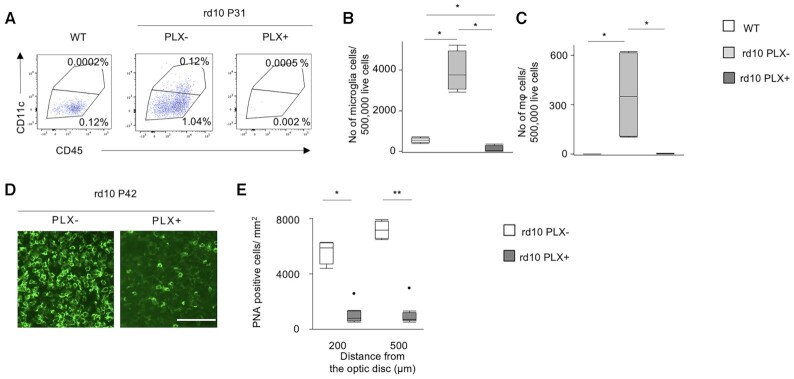
Microglia depletion induced by the CSF1R inhibitor PLX5622 exacerbated cone photoreceptor cell death in rd10 mice. (**A**) Flow cytometry analysis of the retinal samples of rd10 mice fed with or without PLX5622 at P31. WT retinas without treatment were used as control samples for gating microglia and mφ. (**B**) and (**C**) Differences in the number of retinal microgila cells (B) and retinal mφ (C) per 500,000 analyzed cells among WT and rd10 mice fed with or without PLX5622 (all *n* = 4). Both microglia and mφ were mostly depleted in the rd10 mouse retinas 7 days after PLX5622 treatment. (**D**) PNA staining and (**E**) the quantification of PNA-positive cone photoreceptor cells in the retinas of P42 rd10 mice fed with or without PLX5622 (*n* = 4, respectively). Scale bar: 50 μm. The cone density was markedly decreased in the rd10 mice treated with PLX5622. The central horizontal bars indicate the medians, boxes indicate 25th–75th percentiles, and whiskers indicate 1.5 times the interquartile range from the bottom and the top of the box. Wilcoxon rank-sum tests were performed to assess the significance. *P < 0.05, **P < 0.01.

We next assessed the role of microglia by using a CSF1R inhibitor. CSF1R is a receptor essential for microglial signaling and survival (36). Administration of the small molecule CSF1R inhibitor PLX5622 depleted retinal microglia within 7 days (37, 38), but PLX5622 has also been shown to deplete peripherally engrafted monocytes in the retina (3) and tissue-resident macrophages of the lung, liver, peritoneum, and bone marrow (39, 40). We treated WT mice with PLX5622-containing chow from P21, and found that the microglia were mostly depleted in the P31 retina (Figure S5A–C, Supplementary Material). Microglia depletion did not affect the survival of rod or cone cells in the WT mice (Figure S5D–I, Supplementary Material). However, in the rd10 mice, treatment with PLX5622 resulted in depletion of microglia as well as mφ at P31 (Figure 4A–C; Figure S6A–D, Supplementary Material). The cone cell density was markedly decreased in P42 rd10 mice treated with PLX5622 chow compared with the controls (*P* < 0.05; Figure 4D and E). Considering the detrimental effect of peripherally engrafted mφ on cone cells, our data suggest that resident microglia protect the cone cells against degeneration in rd10 mice.

### Inhibition of IMo/mφ protects cone photoreceptors in rd10 mice

**Fig. 5.**
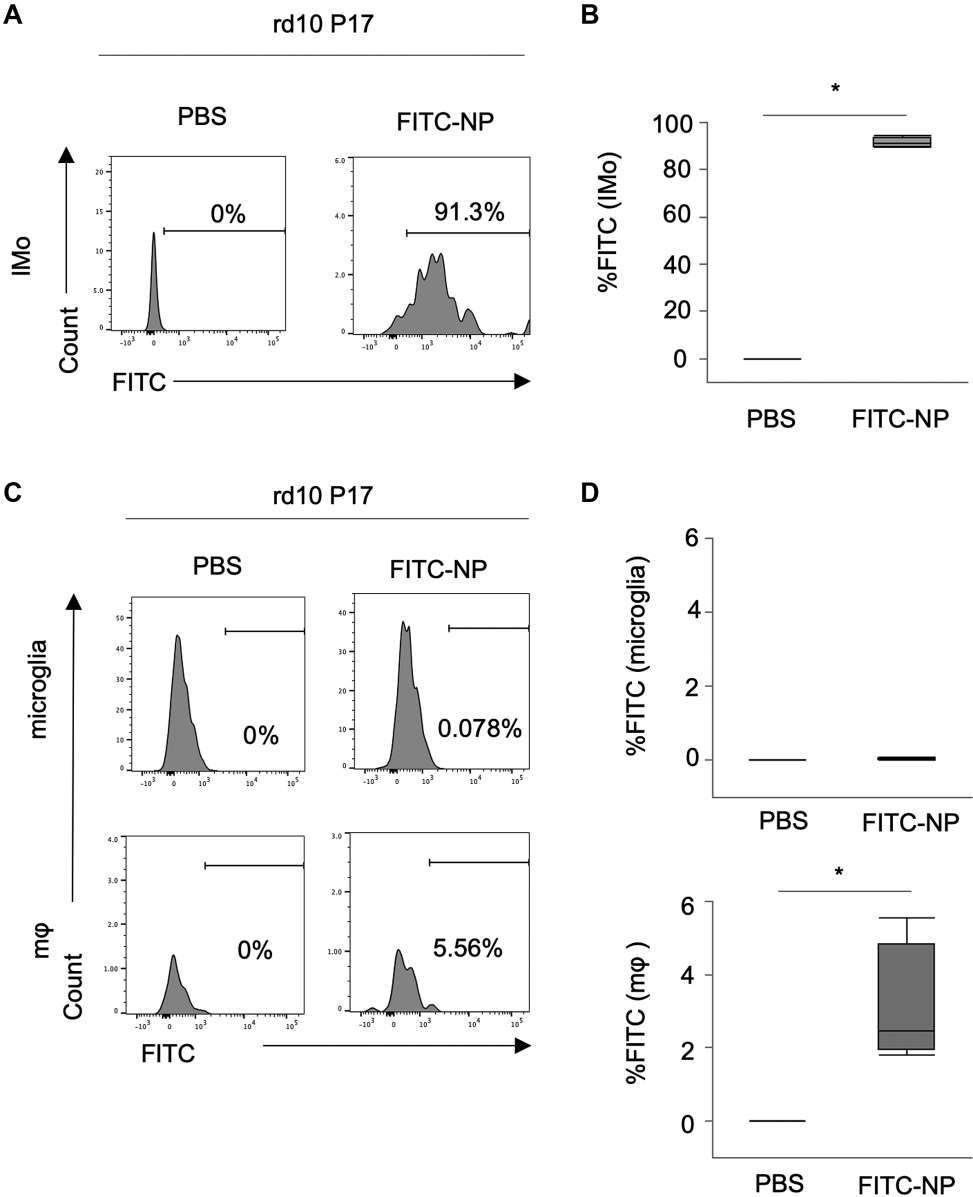
NPs were efficiently incorporated into IMo and retinal mφ in rd10 mice. (**A**) and (**B**) Flow cytometry analysis of the blood samples of P17 rd10 mice administered NPs via the tail vein. Mice were divided into a PBS group (*n* = 4) and an NPs (FITC-NP) group (*n* = 4). (A) Representative data of the FITC incorporation into IMo at 2 h after an injection of PBS and FITC-NPs. (B) The quantification of FITC incorporation into IMo, expressed as the percentage above threshold. (**C**) and (**D**) Flow cytometry analysis of the FITC incorporation in retinal samples of P17 rd10 mice after NP administration (PBS: *n* = 4; FITC-NP: *n* = 4). (C) Representative data of the FITC incorporation into microglia and mφ at 24 h after an intravenous administration of PBS and FITC-NPs. (D) Quantification of FITC incorporation into microglia and mφ. Data are the percentage above threshold. The central horizontal bars indicate the medians, boxes indicate 25th–75th percentiles, and whiskers indicate 1.5 times the interquartile range from the bottom and the top of the box. Wilcoxon rank-sum tests were performed to assess the significance. *P < 0.05.

To further assess whether IMo and peripherally engrafted mφ could be therapeutic targets in RP, we developed a drug delivery method with poly lactide-co-glycolide (PLGA) nanoparticles (NPs) that target IMo, but not microglia. The specificity of the drug delivery method was assessed by injecting fluorescein isothiocyanate (FITC)-loaded NPs into the tail vein of P17 rd10 mice, and the FITC delivery into Ly-6C^hi^ IMo was analyzed at 2 h postinjection. FITC-NPs efficiently introduced FITC into a substantial number of IMo (*P* < 0.05; Figure 5A and B). The drug delivery efficiency to retinal microglia and mφ was analyzed at 24 h after an intravenous injection of NPs (Figure S7A and B, Supplementary Material). FITC-NP administration successfully introduced FITC in 3.1 ± 1.7% of mφ. In contrast, FITC was not detected in microglia by the administration of FITC-NPs (Figure 5C and D). These findings indicate that NPs are a potential drug delivery system for targeting IMo and peripherally engrafted mφ but not for targeting microglia.

**Fig. 6.**
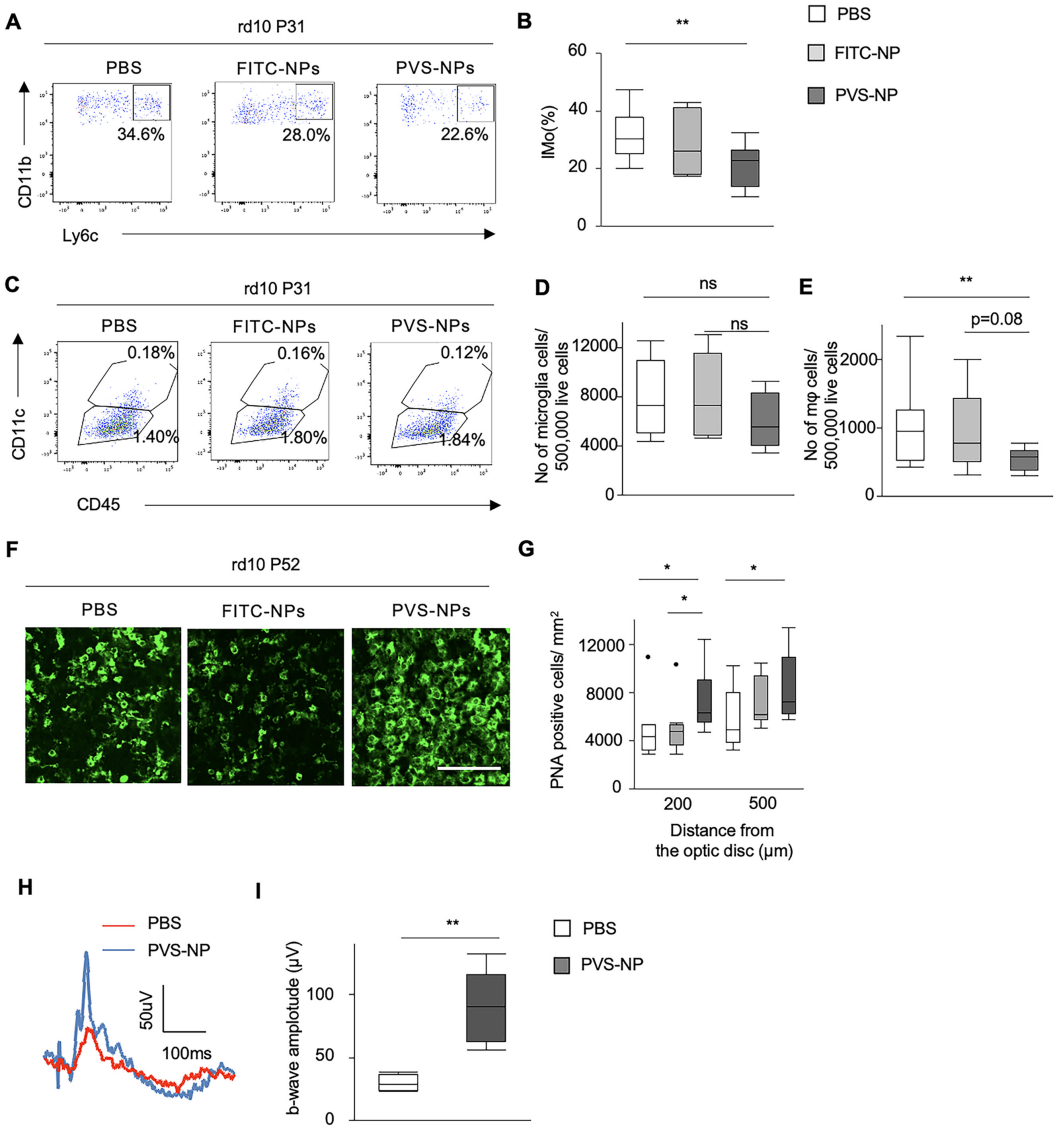
Attenuation of IMo/mφ suppresses cone cell death in rd10 mice. (**A**) Flow cytometry analysis of the blood samples of P31 rd10 mice treated with PBS, FITC-NPs, or PVS-NPs. The mice received an intravenous injection of 1 of these 3 agents 2×/week beginning on P21. The gate was defined as CD11b^+^Ly-6C^hi^Ly-6G^lo−neg^ IMo. (**B**) Changes in the proportion of IMo in the 3 groups (PBS: *n* = 10; FITC-NP: *n* = 11; and PVS-NP: *n* = 14). (**C**) Flow cytometry analysis of the retinal samples of P31 rd10 mice treated with PBS, FITC-NPs, or PVS-NPs. The cells surrounded by the lower gate were defined as microglia, and the cells surrounded by the upper gate were defined as mφ. (**D**) and (**E**) The difference in the number of retinal microglia (D) and mφ (E) per 500,000 analyzed cells among the 3 groups (PBS: *n* = 14; FITC-NP: *n* = 12; and PVS-NP: *n* = 14). (**F**) PNA staining and (**G**) quantification of PNA-positive cone cells in the retina of P52 rd10 mice treated with PBS (*n* = 9), FITC-NP (*n* = 9), or PVS-NP (*n* = 9). Scale bar: 50 μm. (**H**) Photopic ERG and (**I**) the quantification of b-wave amplitudes of P35 rd10 mice treated with PBS (*n* = 6) and rd10; *Ccl2*^−/−^ mice (*n* = 8). The central horizontal bars indicate the medians, boxes indicate 25th–75th percentiles, and whiskers indicate 1.5 times the interquartile range from the bottom and the top of the box. Outliers are shown as dots. Wilcoxon rank-sum tests were performed to assess the significance. *P < 0.05, **P < 0.01.

Pitavastatin-encapsulated PVS-NPs have been used by Katsuki et al. to substantially reduce Ly-6C^hi^ IMo in a mouse model of atherosclerosis (41). We, therefore, evaluated the efficacy of PVS-NPs in rd10 mice. We treated rd10 mice with phosphate-buffered saline (PBS), FITC-NPs, or PVS-NPs, which were intravenously administered 2×/week starting on P21. At P31, blood IMo and retinal mφ were significantly decreased in the PVS-NPs group compared with the PBS group (Figure 6A–D). The proportions of microglia were not significantly different among the 3 treatment groups (Figure 6C and E). The detailed information including the percentages and absolute numbers of microglia/mφ has been demonstrated in Table S3 (Supplementary Material). The cone density at P52 was significantly preserved in the PVS-NP group compared with the PBS and FITC-NP groups (Figure 6F and G). Functional analysis confirmed that the b-wave amplitudes of photopic ERG were significantly higher in the PVS-NP group compared with the PBS group (*P* < 0.01; Figure 6H and I). These results further support that targeting of IMo/mφ is a potent therapeutic strategy to delay cone cell death in rd10 mice.

## Discussion

Accumulating evidence shows that microglial cells are markedly increased and activated in the RP retina (5, 42), but their origin(s) —resident microglia and/or peripherally engrafted mφ —have not been fully elucidated. Although engrafted Mφ resemble microglia in their morphology and longevity, these cells retain a different signature by expressing different levels of surface markers (e.g. CD45, CD11c, and I-A/I-E) and inflammatory molecules (e.g. IL-1β and TNF-α) (3). Using a flow cytometry method to distinguish microglia and mφ in the retina, we demonstrated that the populations of both cell types were increased in rd10 mice. We also showed that CCL2, which are highly upregulated in the eyes of the RP-model mice and human patients (4, 20, 23), are critical for recruitment of CCR2^+^ IMo into the diseased loci. Otani et al. previously reported that CCL2/CCR2 blockade alleviates photoreceptor cell death in RP mice (24), and here we expand this finding by showing that depletion of *Ccl2* in rd10 mice decreases IMo/mφ recruitment into the retina, preserves cone cells, suppresses neuroinflammatory molecules involved in myeloid cell activation (*Snca* and *Tcirg1*), induction of inflammatory gene expression (*Nfkb1* and *Egr1*), and promotion of cell death/inflammation (*Bnip3*). Consistent with our findings, Paschalis et al. demonstrated in a corneal alkali burn model that CCR2^+^ IMo are precursors of mφ that are permanently engrafted into the retina, and these permanently engrafted mφ produce cytotoxic TNF-α (43). In addition, our study demonstrated that increased percentage of CD14^++^CD16^+^ intermediate monocytes, which express high levels of CCR2, is related with faster loss of cone-mediated central vision in patients with RP. Taken together, these findings suggest that chronic accumulation of peripherally engrafted mφ promotes a toxic neuroinflammatory environment which mediates mutation-independent cone cell death in RP.

Monocyte activation in the peripheral blood has been reported in age-related macular degeneration and diabetic retinopathy, 2 diseases whose etiology involves systemic genes and environmental factors (21, 44, 45). In contrast, the disease loci of RP are generally thought to be restricted to the retina, where the mutant proteins are exclusively expressed. Given the small size of the eye, it would be surprising if peripheral monocytes are activated and contribute to the disease progression in RP. In line with our present data, we previously observed that the serum levels of inflammatory markers such as high-sensitivity C-reactive protein and IL-8 are elevated and correlate with worse visual function in RP patients (46, 47), suggesting that a peripheral inflammatory response may be implicated in cone degeneration in RP. Eandi et al. also reported that activated monocytes–macrophages had a detrimental effect on cone cells in retinal injury and age-related macular degeneration (48). These results may indicate that circulating blood IMo as a novel therapeutic target for retinal degenerations.

PLX5622, a small molecule CSF1R inhibitor, has been widely used to selectively deplete microglia and address specific functions of microglia in the CNS (17, 49–51). However, we here observed that PLX5622 diminishes not only the retinal microglia but also mφ in rd10 mice. In line with our findings, Paschalis et al. recently demonstrated that PLX5622 depletes the peripherally engrafted mφ that repopulate the retina after microglia depletion (3). Moreover, CSF1R inhibition was shown to affect hematopoiesis and the function of mφ (52), and even a short exposure (1 week) to PLX5622 was able to deplete tissue-resident Mφs of the liver, lung, peritoneum, and bone marrow (40). Therefore, when interpreting the results of these studies, the effects of CSF1R inhibition on peripheral immune cells must be considered. In our present study, mφ attenuation improved cone survival, suggesting that the detrimental effect of PLX5622 in rd10 mice is primarily mediated by the microglia depletion. In line with our findings, Zhao et al. showed that a toxin-induced depletion of CX3CR1^+^ microglia/mφ substantially inhibited retinal degeneration in rd10 mice (5). However, it should be noted that toxin-induced depletion models may be impacted by the massive cell death, which can include cytokine storm and astrogliosis (53). The precise function of retina-resident microglia should be addressed in future studies using more elaborate cell-depletion techniques.

Modulation of the imbalance between harmful and beneficial immune responses has been suggested as a potential treatment strategy for retinal degenerative diseases (54). Statins exert a potent immune-modulatory effect in addition to their lipid-lowering effect, and both these effects are augmented with high-dose therapy (55). However, there are potential safety concerns regarding the long-term treatment of high-dose statins, including myopathy and liver enzyme elevation (56). We observed that an approximately 10-fold higher dose of oral PVS compared to that used in clinical practice is required to exert anti-inflammatory and protective effects in rd10 mice (Figure S8A and B, Supplementary Material). In contrast, PVS-NPs were shown to efficiently attenuate IMo activation and suppress retinal degeneration in rd10 mice, while reducing the total dosage of PVS (0.65 mg/kg/week with PVS-NPs vs. 7.0 mg/kg/week with high-dose oral PVS). Our collaborators previously showed that PVS-loaded NPs protect against tissue injury in animal models of atherosclerosis and stroke (41, 57), and their efficacy is now being assessed in clinical trials for critical limb ischemia and pulmonary hypertension. The present results suggest that RP may be an alternative target for this NP-loaded statin treatment.

Gene therapy represents a next-generation treatment for RP (8, 9), but its application is currently limited to few genetic mutations, and RP patients who received gene supplementation still show progression of the disease even at the vector-injected site (58). In modern societies, cone-mediated vision, not rod-mediated vision, is critical for the quality of life. Thus, RP patients become aware of limitations when their cone-mediated vision is affected. For this reason, cone preservation therapy by immune modulation may be a clinically important intervention in addition to individualized gene therapy.

## Conclusion

In conclusion, the results of our present study demonstrate that in contrast to resident microglia, IMo and peripherally engrafted mφ promote cone cell death in RP, and their inhibition could be therapeutically achieved through NP administration of statins. The implementation of such therapy in clinical practice may help salvage cone cells and preserve daylight vision in RP patients.

## Materials and Methods

### Animals

WT (C57BL/6J) mice, B6.CXB1-*Pde6β*^rd10^/J (rd10) mice, and B6.129S4-*Ccl2^tm1Rol^*/J (*Ccl2*^−/−^) mice were purchased from The Jackson Laboratory (West Grove, PA). Rd10 mice were crossed with *Ccl2*^−/−^ mice to generate rd10; *Ccl2*^−/−^ mice. Rd10 mutation in the *Pde6b* gene was detected by polymerase chain reaction (PCR) with the use of the forward primer 5′-ATGTAC TCTGCTCCCCAGGT-3′, the reverse primer 5′-GCATCCTTGGGCCAGAAGAT-3′, and subsequent direct DNA sequencing using a Hitachi 3500xl Genetic Analyzer (Applied Biosystems, Foster City, CA). The *Ccl2* genotype was analyzed by PCR using the forward primer 5′-TGACAGTCCCCAGAGTCACA-3′ and the reverse primer 5′-TCATTGGGATCATCTTGCTG-3′ to detect the WT allele, and the forward primer 5′-GCCAGAGGCCACTTGTGTAG-3′ and the reverse primer 5′-TCATTGGGATCATCTTGCTG-3′ to detect the *Ccl2* allele. Both male and female mice were used for the experiment.

### Patients and control subjects

Because blood monocytes can be affected by systemic disorders such as cardiovascular diseases, hypertension, and diabetes (59), we included systemically healthy and relatively young subjects < 45-y-old. Typical RP patients were diagnosed based on the patient's history of night blindness, characteristic fundus findings (e.g. bone spicule-like pigment clumping in the mid-peripheral and peripheral retina), visual field constriction and/or ring scotoma, and reduced or nonrecordable a- and b-wave amplitude on electroretinography. Patients with cone dystrophy, cone-rod dystrophy, Bietti crystalline retinopathy, uveitis, or systemic diseases were excluded. Participants were consecutively recruited in 2017 and 2018, and blood samples were collected from 31 RP patients and 16 age- and gender-matched healthy subjects. The RP patients were followed up > 1 year, and underwent HFA10-2 tests at least 3 times to obtain the MD slope. For the analysis, the subject's visual acuity at the time of blood collection, the HFA10-2 test result at the closest time to the blood collection, and the MD slope were used. The baseline characteristics of all subjects are summarized in Table 1. The examination results of the right eye of each subject were used for analysis. We previously performed a genetic analysis of 83 known RP causative genes, and 27 of the 31 RP patients in our present investigation underwent a genetic test (60). The genetic inheritance patterns were determined based on the detected variants.

**Table 1.** Baseline characteristics of the control subjects and RP patients.

	Control subjects	RP patients	*P-*value	RP causative gene*, *n*
	(*n* = 16)	(*n* = 31)		
Age, years	34.4 ± 3.2	36.3 ± 5.8	0.71	
(range)	(28–42)	(25–45)		
Males : females	6:10	17:14	0.35	
VA, logMAR		0.14 ± 0.36		
MD value, dB		−14.7 ± 9.9		
MD slope, dB/year		−0.43 ± 0.52		
Inheritance mode, *n*				
AD		3		*RHO*, 1; *IMPDH1*, 1; *SNRNP200*, 1
AR		2		*EYS*, 1; *USH2A*, 1
XL		1		*RPGR*, 1
ND		21		
NT		4		

Data are mean ± SD. *These genes were determined in the previous genomic sequence analysis of 83 RP causative genes (60). Bold values: statistically significant at *P* < 0.05. RP, retinitis pigmentosa; VA, visual acuity; logMAR, logarithm of the minimum angle of resolution; MD, mean deviation; dB, decibel; AD, autosomal dominant; AR, autosomal recessive; XL, X-linked; ND, not determined; and NT, not tested.

### Clinical examinations

The subjects' best corrected visual acuity (BCVA) was measured with a Landolt decimal VA chart (CV-6000; Tomey, Nagoya, Japan) at 5 m or with single Landolt test cards (HP-1258; Handaya, Tokyo), and the values were converted to the logarithm of the minimum angle of resolution (logMAR) units. The refractive error was corrected at each visit using multiple lenses with different diopters to confirm that the VA was best-corrected. The VA was based on the minimum Landolt C letter that the subject was able to correctly answer > 60% (3/5) of the time. Automated static perimetry tests were performed with a Humphrey Field Analyzer (HFA; Humphrey Instruments, San Leandro, CA) using the central 10–2 Swedish Interactive Thresholding Algorithm Standard Program. The lens was corrected as appropriate for the test distance. Visual field testing was repeated if the test reliability was not satisfactory (i.e. fixation loss > 20%,  false positive > 15%,  or false negative > 33%).

### Flow cytometry

Immunolabeled cells were analyzed by the BD FACSVerse system (BD Biosciences, Franklin Lakes, NJ) using FlowJo software. The samples were prepared and assayed as follows.

#### Mouse blood monocytes

Peripheral blood was drawn from the mouse via a cardiac puncture, and red blood cells were lysed with VersaLyse Lysing solution (Becton Dickinson Biosciences, San Jose, CA) for 10 min at room temperature (RT). The cells were washed twice in ice-cold fluorescence-activated cell sorting (FACS) buffer, i.e. PBS with 2% fetal bovine serum (FBS). Fc receptors were blocked with anti-mouse CD16/CD32 (eBioscience, Waltham, MA) for 5 min at 4°C, followed by incubation for 20 min on ice with antibodies against mouse CD192 (CCR2; Alexa Fluor 647-conjugated, clone SA203G11; Biolegend, San Diego, CA), CD11b (BV421-conjugated, clone M1/70; Biolegend), Ly-6C (Alexa fluor 488-conjugated, clone HK1.4; Biolegend), Ly-6G (allophycocyanin [APC]-cy7-conjugated, clone 1A8; Biolegend), and CX3CR1 (phycoerythrin [PE]-conjugated, clone SA011F11; Biolegend). Dead cells were excluded with the fluorescent marker 7-AAD (BD Pharmingen, San Diego, CA). IMos were identified as CD11b^+^Ly-6C^hi^Ly-6G^lo−neg^ cells.

#### Mouse microglia and mφ

The mouse retina was harvested from enucleated eyes, minced and digested (with 1.2 mg/ml collagenase D; Roche Diagnostics, Indianapolis, IN and 40 μg/ml DNase I; Sigma-Aldrich, St. Louis, MO) in a water bath at 37°C for 30 min. Following digestion, the tissue was dissociated into single-cell suspensions by pipetting. The cells were washed twice in ice-cold FACS buffer (PBS with 2% FBS). Fc receptors were blocked with anti-mouse CD16/CD32 (eBioscience) for 10 min at 4ºC, followed by incubation for 20 min on ice with antibodies against mouse CD11c (PE-Cy7-conjugated, clone N418; Biolegend), CD45 (APC-conjugated, clone 30-F11; Biolegend), Ly-6C (APC-cy7-conjugated, clone HK1.4; Biolegend), Ly-6G (APC-cy7-conjugated, clone 1A8; Biolegend), CD11b (PE-conjugated, clone M1/70; Biolegend), CD192 (CCR2) (FITC-conjugated, clone SA203G11; Biolegend), CX3CR1 (BV421-conjugated, clone SA011F11; Biolegend), I-A/I-E (BV421-conjugated, clone M5/114.15.2; Biolegend), and F4/80 (FITC-conjugated, clone; BM8 Biolegend). Dead cells were excluded with 7-AAD (BD Pharmingen).

Microglia were defined as CD11b^hi^CD11c^mid^CD45^mid^Ly-6G^lo^Ly-6C^lo^cells, and mφ were defined as CD11b^hi^CD11c^hi^CD45^hi^Ly-6G^lo^Ly-6C^lo^cells, based on the report by O'Koren et al. WT retinas were used as a control for gating microglia and mφ at each experiment (Figure S2A–D, Supplementary Material). The numbers of microglia/mφ in 500,000 analyzed cells in the retina are calculated according to a previous paper (61).

#### Human blood monocytes

Whole blood (8 ml) was obtained from an antecubital vein of each subject by using a BD Vacutainer CPT Cell Preparation Tube with Sodium Heparin (BD Biosciences). Once obtained, the samples underwent immediate (within < 30 min) density gradient separation of mononuclear cells. The separated mononuclear cells were then pelleted with low-speed centrifugation (200 *g*) and aliquoted into 5 ml tubes in PBS with 2% FBS. The samples were stored at −80°C in a freezer until analyzed, and thawed 30 min before the analyses.

For immunolabeling, cells were washed twice in ice-cold FACS buffer (PBS with 2% FBS). Fc receptors were blocked with antimouse CD16/CD32 (eBioscience) for 5 min at 4ºC, followed by incubation for 20 min on ice with antibodies against human CD56 (NCAM; PerCP/Cy5.5-conjugated, clone HCD56; Biolegend), CD2 (PerCP/Cy5.5-conjugated, clone RPA-2.10; Biolegend), CD19 (PerCP/Cy5.5-conjugated, clone HIB19; Biolegend), HLA-DR (APC-conjugated, clone L243; Biolegend), CD14 (PE-conjugated, clone M5E2; Biolegend), CD16 (BV421-conjugated, clone 3G8; Biolegend), CD192 (CCR2; FITC-conjugated, clone K036C2; Biolegend), and CX3CR1 (PEcy7-conjugated, clone 2A9-1; Biolegend). Dead cells were excluded with 7-AAD (BD Pharmingen). Monocytes were identified as HLA-DR^+^CD2^−^CD19^−^CD56^−^ cells. These cells were classified into 3 subsets according to the levels of CD14 and CD16 expression as described previously (62).

### Retinal whole-mount staining

Mouse eyes were enucleated and fixed with 4% paraformaldehyde (PFA) for 1 h at 4°C. After removal of the cornea and lens, the retinas were dissected from the posterior eye cup. Each retina was blocked for 1 h with PBS containing 10% nonfat dried milk and 0.3% Triton-X 100 (9002–93–1; Wako; Osaka, Japan), and then incubated with rabbit anti–Iba-1 antibody (1:100,019–19741, Wako) and FITC-conjugated PNA (1:100, L7381; Sigma-Aldrich) at 4°C overnight. After being washed with PBS, the retinas were incubated with appropriate Alexa Fluor 488- or Alexa Fluor 647-conjugated secondary antibodies (Invitrogen) for 1 h at RT and then mounted on slides. Immunofluorescence images were acquired using a fluorescence microscope (BZ-X700; Keyence, Osaka, Japan). The numbers of PNA^+^ cone photoreceptor cells and Iba-1^+^ microglia/mφ cells were counted in 0.015625-mm^2^ retinal areas in the superior, inferior, temporal, and nasal areas located 250 and 500 μm from the optic disc by using Image J ver. 1.52a software (U.S. National Institutes of Health; NIH), and each number was averaged at both the 250 and 500 μm distances. The names and conditions of the samples were masked from the observers.

### Histologic examination

Mouse eyes were enucleated, fixed with 4% PFA in PBS for 24 h, and then mounted in paraffin. Sections (5 μm thick) were prepared along the horizontal meridian. The sections were subsequently stained with hematoxylin and eosin (H&E). A total of 5 sections were randomly selected from each eye. The number of cells in the ONL was counted in a 100 μm^2^ square area in the central region (200 μm from the optic nerve), mid-peripheral region (500 μm from the optic nerve), and peripheral region (1000 μm from the optic nerve) of the retina in the nasal hemisphere. The tissue samples were assigned numbers and letters, and the conditions were masked from the observers.

### TUNEL staining

TUNEL (terminal deoxynucleotidyl transferase dUTP nick end labeling) staining was performed using an ApopTag Fluorescein In Situ Apoptosis Detection Kit (Merck Millipore, Darmstadt, Germany) according to the manufacturer's instructions. Immunofluorescence images were acquired using a fluorescence microscope (BZ-X700; Keyence), and the numbers of TUNEL-positive cells in 10,000-μm^2^ areas in the central region (200 μm from the optic nerve), mid-peripheral region (500 μm from the optic nerve), and peripheral region (1000 μm from the optic nerve) of the retina in the nasal hemisphere were counted by using Image J software, ver. 1.52a. The ONL areas in each square area were measured, and the density of TUNEL-positive cells in the ONL was calculated and is expressed as cells/mm^2^. The names and conditions of the samples were masked from the observers.

### ERG

Photopic ERG were recorded through LED contact lenses using a PuREC system (PC-100; Mayo Corporation, Aichi, Japan). Mice were anesthetized with an intraperitoneal injection of ketamine (100 mg/kg) and xylazine (10 mg/kg), and the body temperature was maintained at 37°C with a heating pad. The pupils were dilated with 0.5% tropicamide and 0.5% phenylephrine hydrochloride. After topical oxbuprocaine application, LED contact lenses were attached on the mouse cornea. A reference electrode was placed on the tongue and a ground electrode was clipped to the tail. Mice were adapted for 10 min to a background of white light at an intensity of 30 cd/m^2^. A total of 16 photopic flashes were taken at 3.0 cd·s/m^2^ and averaged.

### mRNA profiling in retinal tissue using the Nanostring nCounter platform

The retinal tissue mRNA expressions were profiled and analyzed with the Nanostring nCounter Mouse Neuroinflammation panel, which contains 757 neuroinflammation-related mouse genes and 13 internal reference controls (#115000237; Nanostring Technologies, Seattle, WA). Briefly, the total RNA from a single retina was extracted using the RNeasy kit (Qiagen, Hilden, Germany). A total of 100 ng of RNA in a volume of 5 μl was hybridized to the capture and reporter probe set for 16 h at 65°C according to the manufacturer's instructions. The individual hybridization reactions were washed and eluted per the protocol at the Biomedical Center at Takara Bio (Kusatsu, Japan), and the data were collected using an nCounter Digital Analyzer (Nanostring). The generated data were evaluated using an internal quality control process, and the resulting data were normalized to the geometric mean of the housekeeping genes using the nSolver 4.0 and Advanced Analysis 2.0 software (Nanostring).

We analyzed retinas from female WT mice (C57BL/6J),  rd10 mice, and rd10; *Ccl2*^−/−^ mice at P31, each comprising 3–4 biological repeats. DEGs were defined as genes demonstrating a significant difference in expression at the level of *P* < 0.05 (adjusted *P*-value by *t*-test). Unsupervised hierarchical clustering and a heat map analysis were performed using the nSolver software.

### CSF-1R inhibition

The CSF-1R inhibitor PLX5622 was incorporated into the mouse chow (1,200 ppm formulated in the AIN-76A standard rodent diet; Research Diets, New Brunswick, NJ, provided by Plexxikon, Berkeley, CA). WT or rd10 mice were fed the control chow (AIN-76) or the chow containing PLX5622 from P21 until the end of the experiment.

### Preparation of PLGA NPs

PLGA polymer with an average molecular weight of 20,000 and a lactide-to-glycolide copolymer ratio of 75:25 (Wako Pure Chemical Industries, Osaka, Japan) was used to prepare the NPs. FITC (Dojindo Laboratories, Kumamoto, Japan) or pitavastatin (PVS; Wako) was incorporated into the PLGA NPs.

FITC-NPs were created by using ULREA SS-11 (M Technique Co., Osaka, Japan). First, a tank containing liquid A (an aqueous solution containing 2.0% PVA) was pressurized to 0.3 MPa and then transferred at a set value of 43°C (measured value: approximately 40°C) and a rate of 120 ml/min. Then, liquid B (a solution containing PLGA, FITC, acetone, and ethanol in a weight ratio of 4.04:0.20:63.84:31.92 for FITC-NPs; and a solution containing PLGA, pitavastatin, acetone, and ethanol in a weight ratio of 4.04:0.44: 63.68: 31.84 for PVS-NPs) was transferred at a set value of 41°C (measured value: approximately 30°C) at 100 ml/min. The liquids A and B were reacted on a spinning disc rotating at 1,000 rpm with back pressure of 0.02 MPa. The solvent in the resultant mixture was removed by distillation using an evaporator. The suspension was then purified to remove excess PVA and the unencapsulated reagent, and powdered by the freeze-drying method.

The FITC-NPs contained 6.8 ± 0.4% (w/v) FITC and the PVS-NPs contained 1.3 ± 0.02% (w/v) PVS. The average diameters were as follows: FITC-NPs, 252 nm; and PVS-NPs, 280 nm.

### In vivo biodistribution of the NPs

Blood samples were collected from P17 rd10 mice 2 h after a single intravenous injection of FITC-NP (0.5 mg PLGA/100 μl PBS), and the FITC incorporation in IMo was analyzed by flow cytometry. The retinas were also analyzed 24 h after an intravenous injection of FITC-NP, and the FITC incorporation in mφ and microglia was analyzed.

The blood cells were labeled with mouse Ly-6C (APC-cy7-conjugated, clone HK1.4; Biolegend) and Ly-6G (PerCP/Cy5.5-conjugated, clone 1A8; Biolegend) and the following antibodies: CD192 (CCR2; Alexa Fluor 647-conjugated, clone SA203G11; Biolegend), CD11b (BV421-conjugated, clone M1/70; Biolegend), and CX3CR1 (PE-conjugated, clone SA011F11; Biolegend). The FITC expression was evaluated in CD11b^+^Ly-6C^hi^Ly-6G^lo−neg^ IMo. For the assessment of the cellular uptake of FITC-NPs, CD192 (CCR2; FITC-conjugated, clone SA203G11; Biolegend) was not used.

The retinal cells were stained as described above, with the following: CD11c (PE-Cy7-conjugated, clone N418; Biolegend), CD45 (APC-conjugated, clone 30-F11; Biolegend), Ly-6C (APC-cy7-conjugated, clone HK1.4; Biolegend), Ly-6G (APC-cy7-conjugated, clone 1A8; Biolegend), CD11b (PE-conjugated, clone M1/70; Biolegend), and CX3CR1 (BV421-conjugated, clone SA011F11; Biolegend)]. The FITC expression in microglia and mφ was then analyzed.

### NP treatment of rd10 mice

We divided rd10 mice into 3 groups at P21: the PBS group (100 μl PBS), the FITC-NP group, and the PVS-NP group (0.5 mg PLGA/100 μl PBS). PBS, FITC-NP, or PVS-NP was administered intravenously via the tail vein 2×/week from P21 until the end of each experiment.

### Statistical analyses

The correlation coefficients between the MD slope in the RP patients' HFA10-2 tests and the percentage of monocyte subsets in the patients were analyzed by Spearman's rank correlation test. Comparisons of the data between pairs of groups were performed using the Wilcoxon rank-sum test. Student's *t*-test was used to define DEGs in the retinal tissue mRNA expression analysis. A *P*-value ≤ 0.05 was accepted as significant. The statistical analyses of the data were performed with JMP1 Pro 13.0.0 software (SAS, Cary, NC).

### Study approval

All mice were treated in accord with the standards of the Association for Research in Vision and Ophthalmology for the use of animals in ophthalmic and vision research. All experimental procedures were approved by the Committee for Animals, Recombinant DNA, and Infectious Pathogens Experiments at Kyushu University (A19-134, 30–104). The clinical study was performed according to the tenets of the Declaration of Helsinki on Biomedical Research Involving Human Subjects. The study protocol was approved by the Institutional Review Board of Kyushu University, and informed consent was obtained from all participating subjects after a thorough explanation of the nature of the study and its possible consequences.

## Supplementary Material

pgac003_Supplemental_Files

## Data Availability

All study data are included in the article and/or supporting information.
