# Supplementary material for: Circulating inflammatory monocytes oppose microglia and contribute to cone cell death in retinitis pigmentosa
Source: PNAS Nexus. 2022 Mar 2;1(1):pgac003. doi: 10.1093/pnasnexus/pgac003 (PMC9075747; doi:10.1093/pnasnexus/pgac003)
Supplement: pgac003_Supplemental_Files [file pgac003_supplemental_files.zip › PNASNEXUS-PNASNEXUS-2021-00163-s01.docx]

Expanded View for

Circulating inflammatory monocytes oppose microglia and contribute to cone cell death in retinitis pigmentosa

Jun Funatsu, Yusuke Murakami, Shotaro Shimokawa, Shunji Nakatake, Kohta Fujiwara, Ayako Okita, Masatoshi Fukushima, Kensuke Shibata, Noriko Yoshida, Yoshito Koyanagi, Masato Akiyama, Shoji Notomi, Shintaro Nakao, Toshio Hisatomi, Atsunobu Takeda, Eleftherios I. Paschalis, Demetrios G. Vavvas, Yasuhiro Ikeda and Koh-Hei Sonoda

***Corresponding author:** Yusuke Murakami, MD, PhD, Department of Ophthalmology, Graduate School of Medical Sciences, Kyushu University, 3-1-1 Maidashi, Higashi-ku, Fukuoka, 812-8582, Japan. Tel.: +81-92-642-5648; Fax: +81-92-642-5663. E-mail: murakami.yusuke.407@m.kyushu-u.ac.jp

Supplementary text

Table S1 to S3

Figures S1 to S8

**Supplementary Material and Methods**

**Oral administration of PVS**

We divided rd10 mice into three groups at P21: the 0.5% carboxymethyl cellulose (CMC) group (100 μl 0.5% CMC/day) as a control, the 0.1 mg/kg PVS group (0.1 mg/kg PVS in 100 μl 0.5% CMC/day), and the 1.0 mg/kg PVS group (1.0 mg/kg PVS in 100 μl 0.5% CMC/day). Each drug was administered daily by oral gavage from P21 until the end of experiment, P52. The PVS was provided by Kowa Pharmaceutical Co., Ltd., Aichi, Japan.

**Retinal whole-mount staining**

Mouse eyes were enucleated and fixed with 4% paraformaldehyde (PFA) for 1 hr at 4°C. After removal of the cornea and lens, the retinas were dissected from the posterior eye cup. Each retina was blocked for 1 hr with PBS containing 10% nonfat dried milk and 0.3% Triton-X 100 (9002-93-1; Wako), and then incubated with FITC-conjugated PNA (1:100, L7381; Sigma-Aldrich) at 4°C overnight. Immunofluorescence images were acquired using a Fluorescence Microscope (BZ-X700; Keyence, Osaka, Japan). The numbers of PNA^+^ cone photoreceptor cells were counted in 0.015625-mm^2^ retinal areas in the superior, inferior, temporal, and nasal areas located 250 μm and 500 μm from the optic disc by using Image J ver. 1.52a software (U.S. National Institutes of Health [NIH]), and each number was averaged at both the 250 μm and 500 μm distances. The names and conditions of the samples were masked from the observers.

**Table S1. The absolute numbers of microglia and mφ as well as the total numbers of live cells in the retinas (wt vs rd10).**

|  | live cells | mφ count | microglia count | mφ/live cells % | microglia/ live cells % |
| --- | --- | --- | --- | --- | --- |
| wt P21 1 | 838000 | 3 | 2906 | 0.000126 | 0.216 |
| wt P21 2 | 574000 | 1 | 2033 | 0.000186 | 0.243 |
| wt P21 3 | 1040000 | 4 | 3576 | 0.000203 | 0.22 |
| wt P21 4 | 642000 | 2 | 2326 | 0.000325 | 0.195 |
| wt P21 5 | 761000 | 2 | 2114 | 0.000284 | 0.184 |
| wt P21 6 | 661000 | 3 | 1896 | 0.000322 | 0.182 |
| wt P21 7 | 764000 | 4 | 2968 | 0.000414 | 0.251 |
| wt P21 8 | 868000 | 3 | 3054 | 0.000247 | 0.222 |
| **average** | **768500** | **2.75** | **2609.12** | **0.000263** | **0.214** |
|  |  |  |  |  |  |
| rd10 P21 1 | 220410 | 259 | 4396 | 0.118 | 1.48 |
| rd10 P21 2 | 311644 | 573 | 9694 | 0.181 | 2.28 |
| rd10 P21 3 | 313602 | 259 | 4338 | 0.0835 | 0.986 |
| rd10 P21 4 | 301737 | 240 | 3912 | 0.0765 | 0.91 |
| rd10 P21 5 | 432000 | 192 | 3450 | 0.04 | 0.586 |
| rd10 P21 6 | 591000 | 191 | 5380 | 0.0306 | 0.592 |
| rd10 P21 7 | 529000 | 195 | 4895 | 0.0359 | 0.652 |
| **average** | **385627.57** | **272.71** | **5152.14** | **0.0807** | **1.069** |
|  |  |  |  |  |  |
| wt P31 1 | 2090000 | 12 | 4298 | 0.000406 | 0.106 |
| wt P31 2 | 2070000 | 8 | 4322 | 0.000309 | 0.102 |
| wt P31 3 | 2110000 | 9 | 4723 | 0.000556 | 0.109 |
| wt P31 4 | 879000 | 1 | 1928 | 0.000154 | 0.121 |
| wt P31 5 | 592000 | 2 | 1411 | 0.000219 | 0.129 |
| wt P31 6 | 742000 | 6 | 2459 | 0.000719 | 0.205 |
| wt P31 7 | 1170000 | 5 | 4583 | 0.000459 | 0.21 |
| wt P31 8 | 512000 | 2 | 1516 | 0.000414 | 0.179 |
| wt P31 9 | 638000 | 4 | 2446 | 0.000847 | 0.226 |
| **average** | **1200333.33** | **5.44** | **3076.22** | **0.000453** | **0.154** |
|  |  |  |  |  |  |
| rd10 P31 1 | 505000 | 443 | 12446 | 0.0856 | 1.21 |
| rd10 P31 2 | 255288 | 145 | 2014 | 0.0452 | 0.656 |
| rd10 P31 3 | 532000 | 504 | 6351 | 0.0897 | 0.944 |
| rd10 P31 4 | 292137 | 275 | 3679 | 0.0843 | 0.981 |
| rd10 P31 5 | 618000 | 262 | 3911 | 0.0393 | 0.462 |
| rd10 P31 6 | 590000 | 141 | 3470 | 0.0272 | 0.436 |
| **average** | **465404.16** | **295** | **5311.83** | **0.0618** | **0.781** |
|  |  |  |  |  |  |
| wt P42 1 | 818000 | 5 | 1437 | 0.000557 | 0.101 |
| wt P42 2 | 1090000 | 3 | 2356 | 0.000202 | 0.129 |
| wt P42 3 | 1220000 | 3 | 3261 | 0.00037 | 0.161 |
| wt P42 4 | 1060000 | 2 | 1900 | 0.000103 | 0.103 |
| wt P42 5 | 1090000 | 6 | 6148 | 0.000388 | 0.192 |
| wt P42 6 | 915000 | 2 | 4993 | 0.000231 | 0.241 |
| wt P42 7 | 843000 | 6 | 3679 | 0.000639 | 0.241 |
| **average** | **1005142.86** | **3.85** | **3396.28** | **0.000355** | **0.166** |
|  |  |  |  |  |  |
| rd10 P42 1 | 340834 | 125 | 4222 | 0.0358 | 0.733 |
| rd10 P42 2 | 260542 | 121 | 5227 | 0.0516 | 0.996 |
| rd10 P42 3 | 168766 | 75 | 2092 | 0.054 | 0.975 |
| rd10 P42 4 | 265520 | 128 | 3272 | 0.0494 | 0.96 |
| rd10 P42 5 | 57553 | 42 | 880 | 0.0784 | 1.32 |
| rd10 P42 6 | 143457 | 67 | 1523 | 0.0468 | 0.875 |
| rd10 P42 7 | 285938 | 125 | 3499 | 0.0433 | 0.902 |
| rd10 P42 8 | 701000 | 146 | 5305 | 0.0214 | 0.485 |
| rd10 P42 9 | 613000 | 91 | 3439 | 0.0148 | 0.382 |
| **average** | **315178.88** | **102.22** | **3273.22** | **0.0439** | **0.847** |
|  |  |  |  |  |  |

**Table S2. Chemokines/receptors related genes that are significantly differentially expressed in rd10 compared to WT are shown.**

|  | **Log2 fold change** | **P-value** |
| --- | --- | --- |
| **Csf1** | 1.75 | 8.16E-06 |
| **Cx3cl1** | 1.3 | 8.18E-06 |
| **Cx3cr1** | 2.93 | 8.11E-06 |
| **Ifnar1** | 0.228 | 0.0115 |
| **Ifnar2** | 1.17 | 2.9E-05 |
| **Il1r1** | 1.63 | 3.59E-05 |
| **Il1rap** | 0.937 | 0.000345 |
| **Il2rg** | 0.676 | 0.0112 |
| **Ltbr** | 1.81 | 8.44E-06 |
| **Ngf** | 0.811 | 0.01 |
| **Ngfr** | 1.16 | 0.000139 |
| **Osmr** | 2.34 | 1.02E-07 |
| **Tgfb1** | 1.58 | 0.000614 |
| **Tgfbr1** | 0.431 | 0.000202 |
| **Tnfrsf1a** | 2.72 | 5.15E-07 |
| **Tnfsf12** | -1.83 | 1.93E-05 |

**Table S3. The absolute numbers of microglia and mφ as well as the total numbers of live cells in the retinas (rd10 P31; pbs vs FITC-NPs vs PVS-NPs).**

|  | live cells | mφ count | microglia count | mφ/live cells % | microglia/ live cells % |
| --- | --- | --- | --- | --- | --- |
| pbs 1 | 239536 | 247 | 2423 | 0.103 | 1.012 |
| pbs 2 | 257717 | 269 | 2575 | 0.104 | 0.999 |
| pbs 3 | 181909 | 155 | 1589 | 0.085 | 0.874 |
| pbs 4 | 175096 | 154 | 1726 | 0.088 | 0.986 |
| pbs 5 | 36425 | 69 | 549 | 0.189 | 1.507 |
| pbs 6 | 83639 | 186 | 1871 | 0.222 | 2.237 |
| pbs 7 | 69530 | 325 | 1744 | 0.467 | 2.508 |
| pbs 8 | 73688 | 241 | 1599 | 0.327 | 2.170 |
| pbs 9 | 75088 | 246 | 1678 | 0.328 | 2.235 |
| pbs 10 | 71932 | 164 | 1441 | 0.228 | 2.003 |
| pbs 11 | 106162 | 190 | 1504 | 0.179 | 1.417 |
| pbs 12 | 81791 | 177 | 1409 | 0.216 | 1.723 |
| pbs 13 | 245548 | 375 | 2582 | 0.153 | 1.052 |
| pbs 14 | 200873 | 380 | 2192 | 0.189 | 1.091 |
| **average** | **135638** | **227** | **1777** | **0.206** | **1.558** |
|  |  |  |  |  |  |
| FITC 1 | 169966 | 190 | 1862 | 0.112 | 1.096 |
| FITC 2 | 186719 | 181 | 1917 | 0.097 | 1.027 |
| FITC 3 | 172916 | 203 | 1632 | 0.117 | 0.944 |
| FITC 4 | 201245 | 272 | 2036 | 0.135 | 1.012 |
| FITC 5 | 117420 | 340 | 2218 | 0.290 | 1.889 |
| FITC 6 | 132714 | 532 | 3203 | 0.401 | 2.413 |
| FITC 7 | 144858 | 255 | 2618 | 0.176 | 1.807 |
| FITC 8 | 115933 | 320 | 2526 | 0.276 | 2.179 |
| FITC 9 | 121257 | 462 | 3163 | 0.381 | 2.609 |
| FITC 10 | 142422 | 341 | 3337 | 0.239 | 2.343 |
| FITC 11 | 127749 | 113 | 1191 | 0.088 | 0.932 |
| FITC 12 | 148476 | 91 | 1377 | 0.061 | 0.927 |
| **average** | **148473** | **275** | **2257** | **0.198** | **1.598** |
|  |  |  |  |  |  |
| PVS 1 | 155709 | 151 | 1724 | 0.097 | 1.107 |
| PVS 2 | 179683 | 138 | 1985 | 0.077 | 1.105 |
| PVS 3 | 168312 | 189 | 1623 | 0.112 | 0.964 |
| PVS 4 | 190832 | 254 | 1922 | 0.133 | 1.007 |
| PVS 5 | 195773 | 257 | 2786 | 0.131 | 1.423 |
| PVS 6 | 132636 | 170 | 2107 | 0.128 | 1.589 |
| PVS 7 | 204790 | 124 | 1412 | 0.061 | 0.689 |
| PVS 8 | 374872 | 276 | 3049 | 0.074 | 0.813 |
| PVS 9 | 123722 | 176 | 2109 | 0.142 | 1.705 |
| PVS 10 | 240815 | 288 | 3930 | 0.120 | 1.632 |
| PVS 11 | 117835 | 184 | 2114 | 0.156 | 1.794 |
| PVS 12 | 195475 | 234 | 3610 | 0.120 | 1.847 |
| PVS 13 | 201142 | 210 | 1520 | 0.104 | 0.756 |
| PVS 14 | 375254 | 236 | 2918 | 0.063 | 0.778 |
| **average** | **204061** | **206** | **2344** | **0.108** | **1.229** |


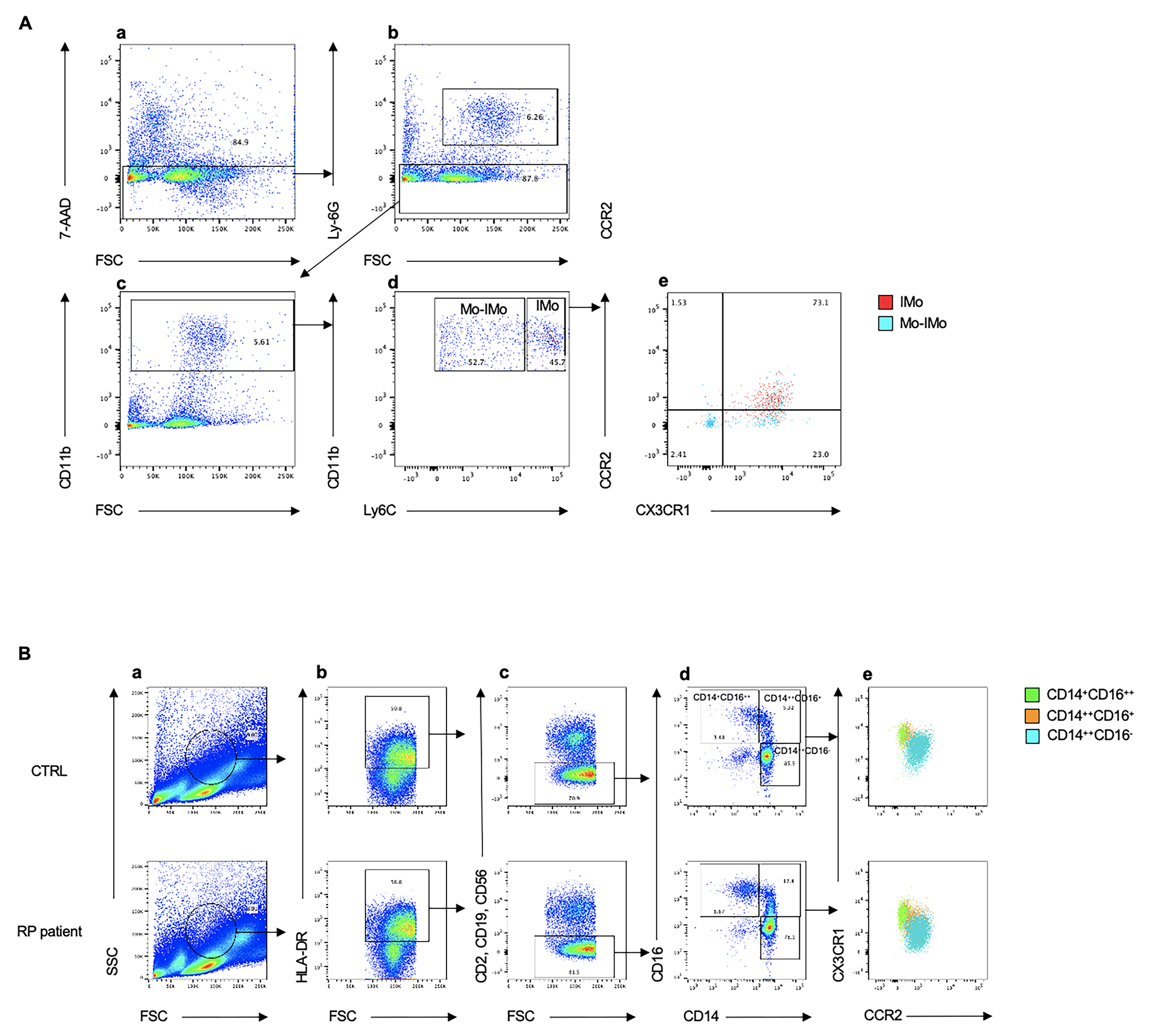


**Fig. S1.** **IMo analysis of the mouse and human peripheral blood samples.** (A) Methods used for the flow cytometry of mouse peripheral blood. The data are from a P21 rd10 mouse sample. (a) Gate on live cells. (b) Gate on Ly-6G^lo−neg^ cells. (c) Gate on CD11b^+^ cells. (d) Gate on Ly-6C^high^ monocytes. (e) Expressions of CCR2 and CX3CR1 of Ly-6C^high^ monocytes (*red dots*) and Ly-6C^lo^ monocytes (*blue dots*). (B) Methods used for the flow cytometry of human peripheral blood. The data are from control and RP patient samples. (a) Gate on monocytes. (b) Gate on HLA-DR^+^ cells. (c) Gate on CD2^−^, CD19^−^, and CD56^−^ cells. (d) Gate on CD14^+^CD16^++^, CD14^++^CD16^−^, and CD14^++^CD16^+^ monocytes. (e) Expressions of CCR2 and CX3CR1 of CD14^+^CD16^++^ (*green dots*), CD14^++^ CD16^−^ (*blue dots*), and CD14^++^CD16^+^ (*orange dots*) monocytes.

**
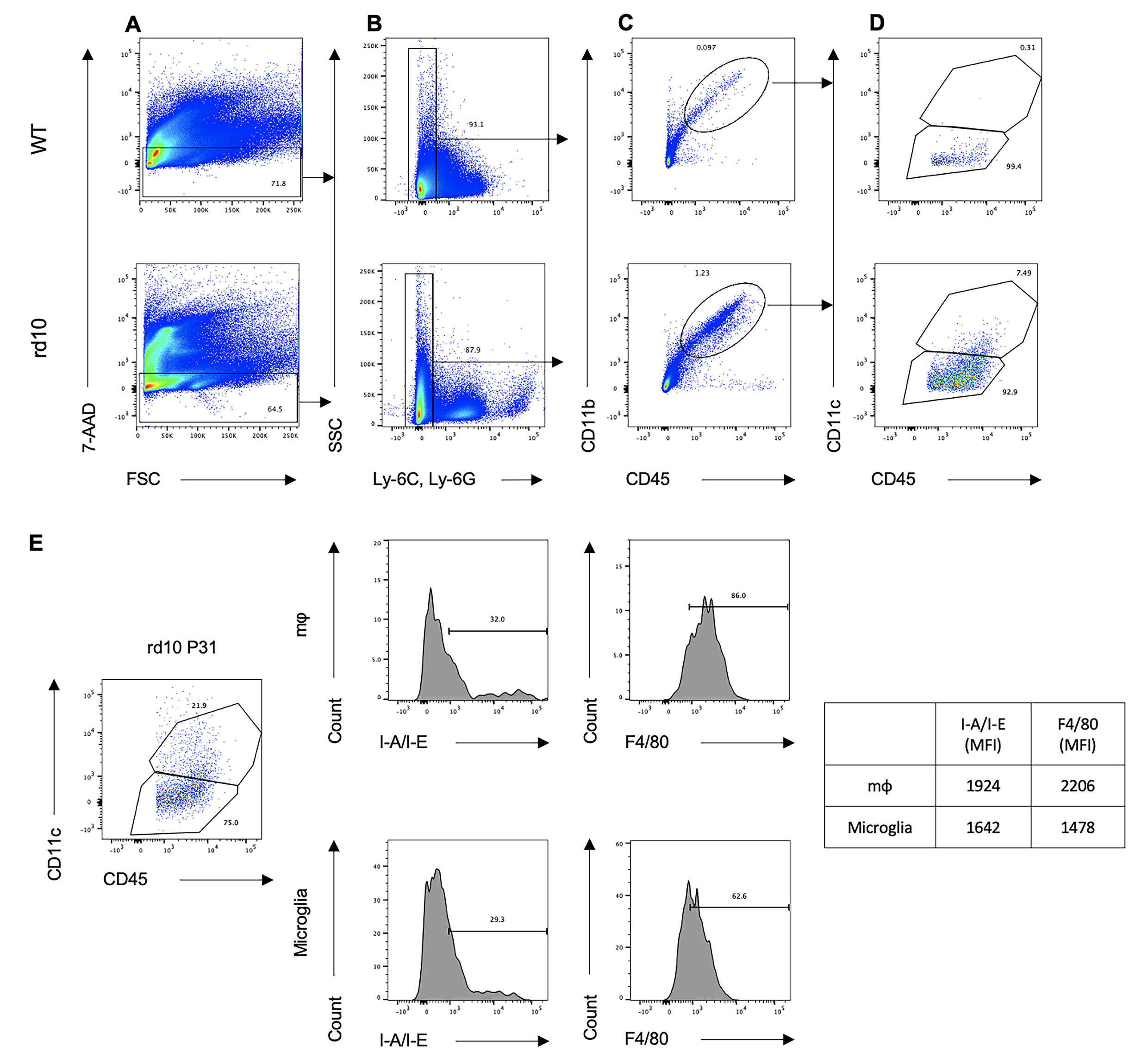
**

**Fig. S2. Flowcytometry analysis of Microglia and mφ in WT and rd10 retinas.** The data are from P31 WT and rd10 mice. (A) Gate on live cells. (B) Gate on Ly-6G^lo−neg^ cells and Ly-6C^lo−neg^ cells. (C) Gate on CD11b^+^CD45^+^ cells. (D) Gate on microglia (lower gate) and mφ (upper gate). (E) Expression levels of F4/80 and MHC class II (I-A/I-E) in microglia (lower gate) and mφ (upper gate).


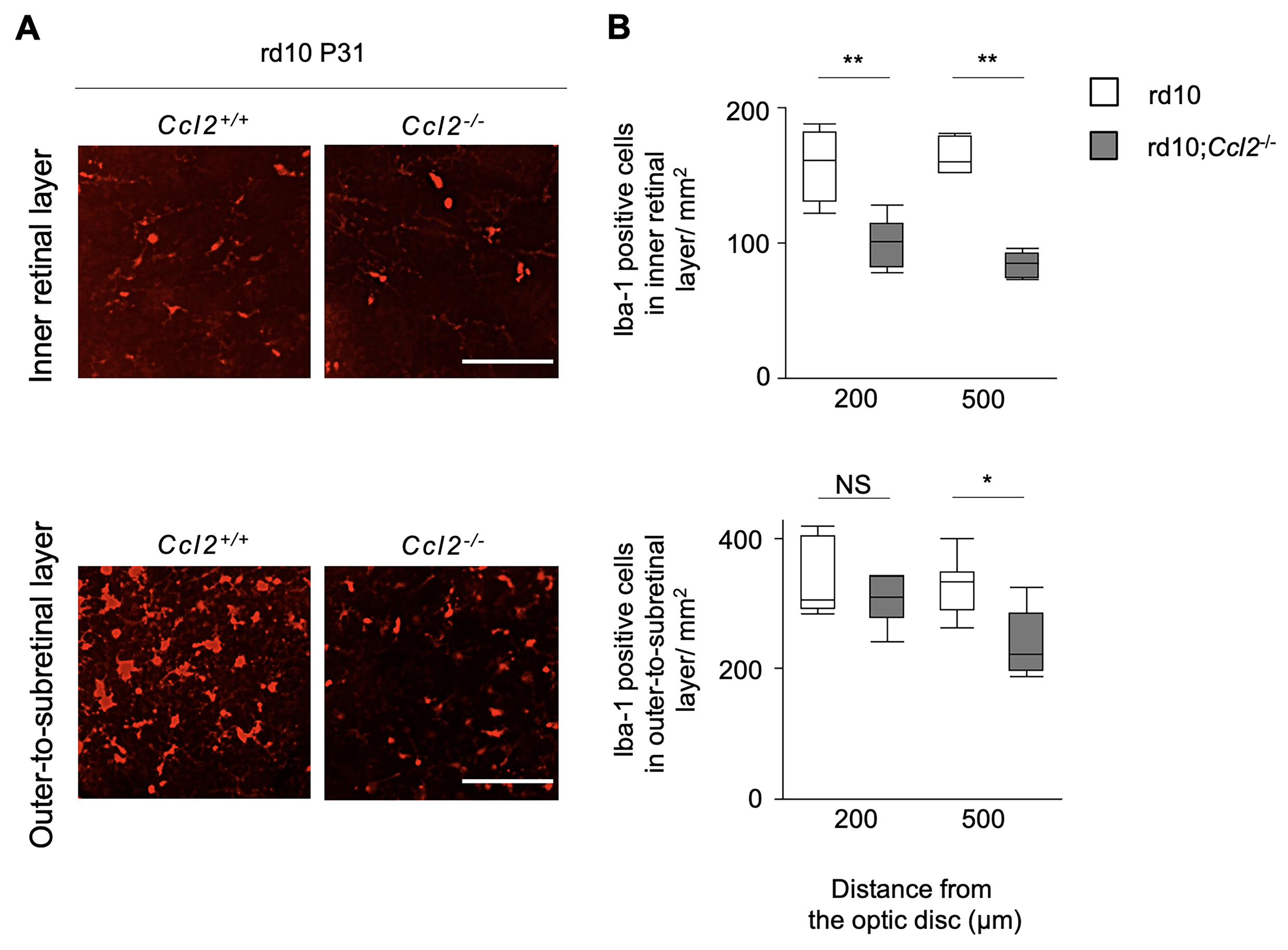


**Fig. S3. Changes in microglia/macrophage distribution by *Ccl2* deficiency.** (**A**) Whole-mount immunostaining for Iba-1 and (**B**) quantification of Iba-1-positive microglia/macrophages in inner or outer-to-subretinal retinal layer of P31 rd10; *Ccl2*^+/+^ (n = 7) and rd10; *Ccl2*^-/-^ mice (n = 6). Scale bar: 50 μm. Note that Iba-1-positive cells were decreased more significantly in the inner retinal layer by *Ccl2* deficiency.

**
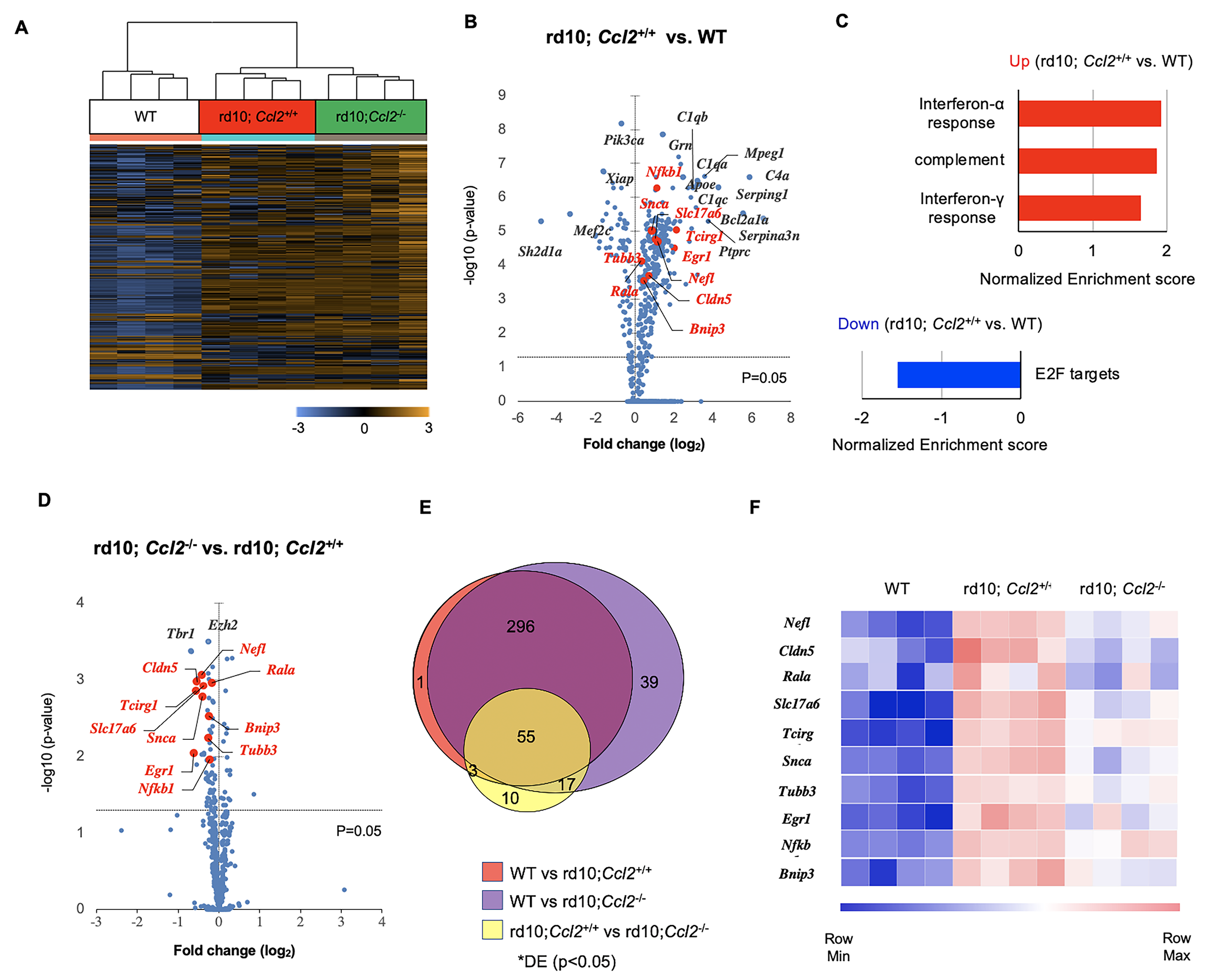
**

**Fig. S4 - Changes in the neuroinflammatory gene profile in *Ccl2*-deficient rd10 retina.** (**A**) Unsupervised clustering of all genes that were expressed above background levels in the WT, rd10; *Ccl2*^+/+^, and rd10; *Ccl2*^−/−^ mouse retina at P31 (all n = 4). (**B**) Volcano plots showing the distributions of DEGs between the WT and rd10; *Ccl2*^+/+^ mouse retina. DEGs were defined as genes showing a significant difference in expression level (p < 0.05). Student's *t*-test was performed to assess significance. (**C**) Hallmark gene sets enrichment scores by GSEA analysis of rd10; *Ccl2*^+/+^ mouse retina compared with WT mouse retina. Gene sets with a family-wise error rate < 0.05 are demonstrated. (**D**) Volcano plots showing the distributions of DEGs between the rd10; *Ccl2*^+/+^ and rd10; *Ccl2*^−/−^ mouse retina. (**E**) Venn diagram visualizing the overlapping results between the DEGs found in the WT vs. rd10, WT vs. rd10; *Ccl2*^−/−^, and rd10 vs. rd10; *Ccl2*^−/−^ comparisons. (**F**) Heat map results showing the top 10 DEGs that were significantly changed in the rd10 vs. WT retina comparison and reversed by *Ccl2* deficiency.


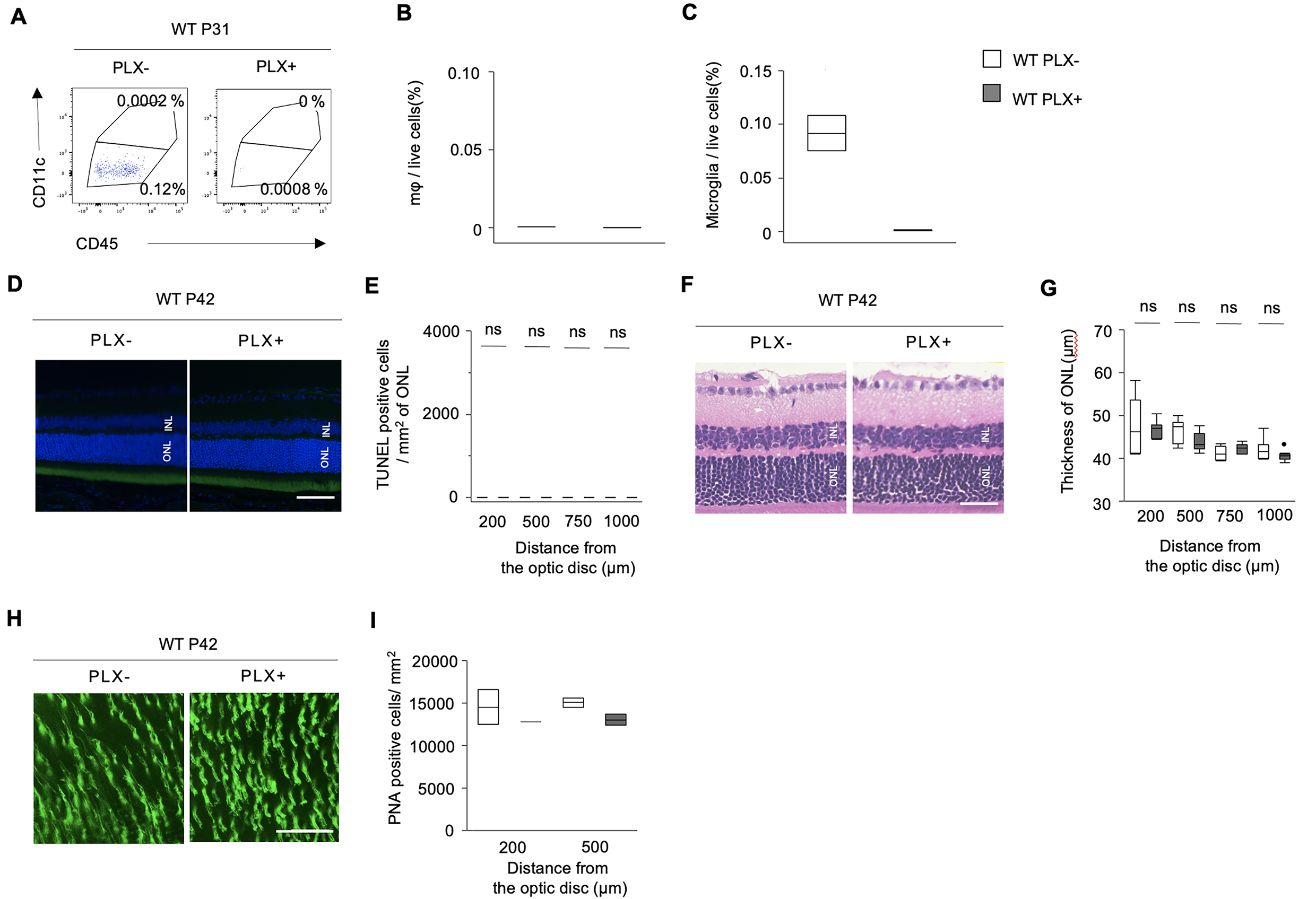


**Fig. S5. Microglia depletion by PLX5622 did not induce retinal toxicity in WT mice.** (A) The retinal samples of WT mice fed the control chow or PLX5622-containing chow were analyzed by flow cytometry at P31. D11b^hi^CD11c^mid^CD45^mid^Ly-6G^lo^Ly-6C^lo^ cells were defined as microglia (lower gate), and CD11b^hi^CD11c^hi^CD45^hi^Ly-6G^lo^Ly-6C^lo^ cells were defined as mφ (upper gate). (B, C) The proportion of microglia (B) and mφ (C) in live cells among WT mice fed with or without PLX5622 (all n = 2). (D) TUNEL staining (green) and (E) quantification of TUNEL-positive cells in the retina of P21 WT mice fed with or without (n = 5, each) PLX5622. Scale bar: 50 μm. (F, G) Histological findings of the retina (F) and quantitative analysis of ONL thickness (G) in the retina of P26 WT mice fed with or without PLX5622 (n = 5, each). Scale bar: 50 μm. (H, I) PNA staining (H) and the quantification of PNA-positive cone cells (I) in the retina of P42 WT mice fed with or without PLX5622 (n = 2, respectively). Scale bar: 50 μm. The central horizontal bars indicate the medians, boxes indicate 25th to 75th percentiles, and whiskers indicate 1.5 times the interquartile range from the bottom and the top of the box. Outliers are shown as dots. Wilcoxon rank sum tests were performed to assess the significance. *p < 0.05, **p < 0.01.


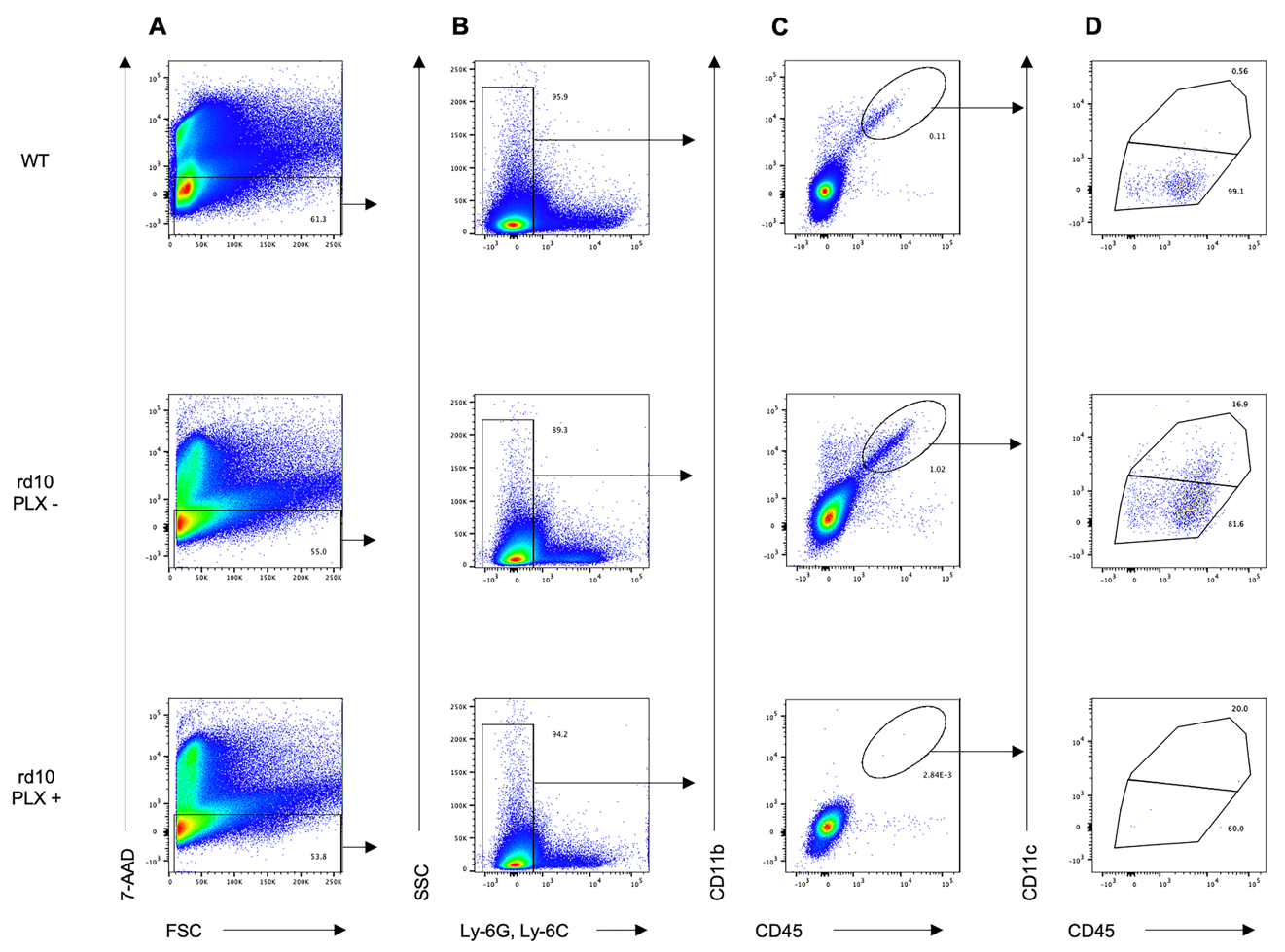


**Fig. S6. Microglia and mφ changes in the rd10 mouse retina after PLX5622 treatment.** Flow cytometry of mouse retinas after PLX5622 treatment. The data are from P31 WT mice, rd10 mice treated with control chow, and rd10 mice treated with PLX5622-containing chow starting from P21. (A) Gate on live cells. (B) Gate on Ly-6G^lo−neg^ cells and Ly-6C^lo−neg^ cells. (C) Gate on CD11b^+^ cells. (D) Gate on microglia (lower gate) and mφ (upper gate).


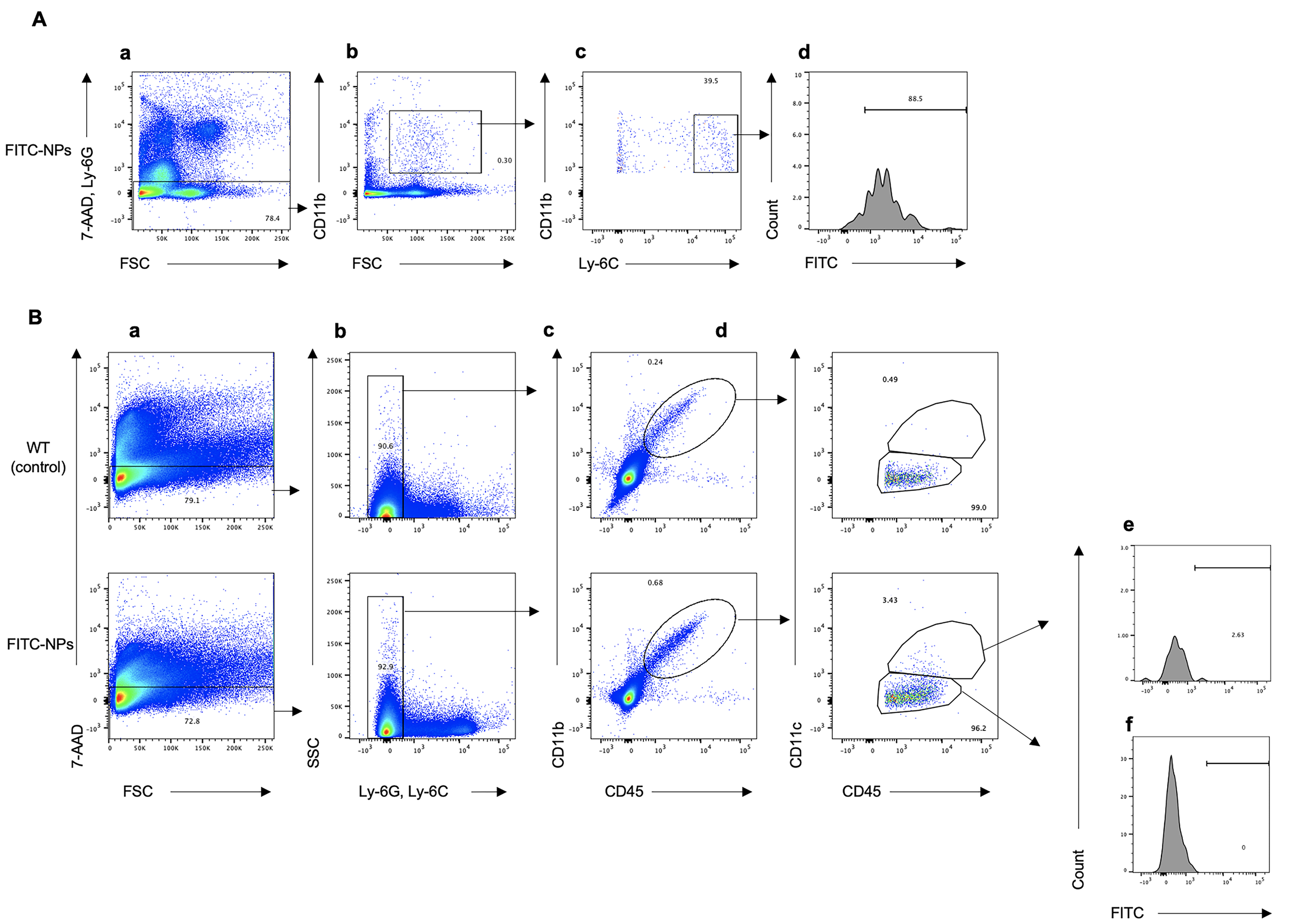


**Fig. S7. Flow cytometry to assess FITC incorporation into the IMo, mφ, and microglia after intravenous NP injection.** (A) Methods used to evaluate FITC incorporation into the IMo. Blood samples were collected from P17 rd10 mice 2 hr after the tail vein administration of FITC-NPs. (a) Gate on live cells and Ly-6G^lo−neg^ cells. (b) Gate on CD11b^+^ cells. (c) Gate on Ly-6C^high^ IMo. (d) The percentage of FITC-incorporated cells in Ly-6C^high^ IMo. (B) Methods used to evaluate FITC incorporation into the mφ and microglia. Retinal samples were collected from P17 WT and rd10 mice 24 hr after a tail vein administration of FITC-NPs. (a) Gate on live cells. (b) Gate on Ly-6G^lo−neg^ cells and Ly-6C^lo−neg^ cells. (c) Gate on CD11b^+^ cells. (d) Gate on mφ (upper gate) and microglia (lower gate). (e, f) The percentage of FITC-incorporated mφ (e) and microglia (f) in rd10 mouse retina. WT retinas were used as controls for gating mφ and microglia.

**
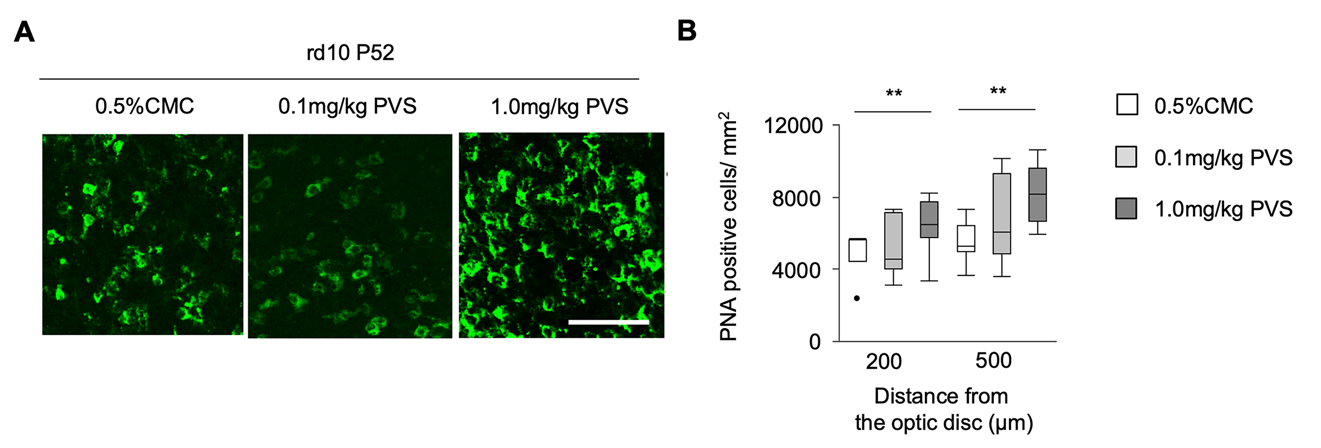
**

**Fig. S8. Oral administration of high-dose statins suppressed cone cell death in rd10 mice.** The rd10 mice were orally administered the control agent (0.5% carboxymethyl cellulose: CMC), 0.1 mg/kg PVS, or 1.0 mg/kg PVS daily from P21 to P52. The mouse retinas were analyzed at P52. (A, B) PNA staining (A) and the quantification of PNA-positive cone cells (B) in the retina of P52 rd10 mice (0.5% CMC: n = 7; 0.1 mg/kg PVS: n = 8; 1.0 mg/kg PVS: n = 11). Scale bar: 50 μm. The central horizontal bars indicate the medians, boxes indicate 25th to 75th percentiles, and whiskers indicate 1.5 times the interquartile range from the bottom and the top of the box. Outliers are shown as dots. Wilcoxon rank sum tests were performed to assess the significance. *p < 0.05, **p < 0.01.
