# Supplementary material for: Circulating inflammatory monocytes oppose microglia and contribute to cone cell death in retinitis pigmentosa
Source: PNAS Nexus. 2022 Mar 2;1(1):pgac003. doi: 10.1093/pnasnexus/pgac003 (PMC9075747; doi:10.1093/pnasnexus/pgac003)
Supplement: pgac003_Supplemental_Files [file pgac003_supplemental_files.zip › PNASNEXUS-PNASNEXUS-2021-00163-s02.docx]

**Table S1. The absolute numbers of microglia and mφ as well as the total numbers of live cells in the retinas (wt vs rd10).**

|  | live cells | mφ count | microglia count | mφ/live cells % | microglia/ live cells % |
| --- | --- | --- | --- | --- | --- |
| wt P21 1 | 838000 | 3 | 2906 | 0.000126 | 0.216 |
| wt P21 2 | 574000 | 1 | 2033 | 0.000186 | 0.243 |
| wt P21 3 | 1040000 | 4 | 3576 | 0.000203 | 0.22 |
| wt P21 4 | 642000 | 2 | 2326 | 0.000325 | 0.195 |
| wt P21 5 | 761000 | 2 | 2114 | 0.000284 | 0.184 |
| wt P21 6 | 661000 | 3 | 1896 | 0.000322 | 0.182 |
| wt P21 7 | 764000 | 4 | 2968 | 0.000414 | 0.251 |
| wt P21 8 | 868000 | 3 | 3054 | 0.000247 | 0.222 |
| **average** | **768500** | **2.75** | **2609.12** | **0.000263** | **0.214** |
|  |  |  |  |  |  |
| rd10 P21 1 | 220410 | 259 | 4396 | 0.118 | 1.48 |
| rd10 P21 2 | 311644 | 573 | 9694 | 0.181 | 2.28 |
| rd10 P21 3 | 313602 | 259 | 4338 | 0.0835 | 0.986 |
| rd10 P21 4 | 301737 | 240 | 3912 | 0.0765 | 0.91 |
| rd10 P21 5 | 432000 | 192 | 3450 | 0.04 | 0.586 |
| rd10 P21 6 | 591000 | 191 | 5380 | 0.0306 | 0.592 |
| rd10 P21 7 | 529000 | 195 | 4895 | 0.0359 | 0.652 |
| **average** | **385627.57** | **272.71** | **5152.14** | **0.0807** | **1.069** |
|  |  |  |  |  |  |
| wt P31 1 | 2090000 | 12 | 4298 | 0.000406 | 0.106 |
| wt P31 2 | 2070000 | 8 | 4322 | 0.000309 | 0.102 |
| wt P31 3 | 2110000 | 9 | 4723 | 0.000556 | 0.109 |
| wt P31 4 | 879000 | 1 | 1928 | 0.000154 | 0.121 |
| wt P31 5 | 592000 | 2 | 1411 | 0.000219 | 0.129 |
| wt P31 6 | 742000 | 6 | 2459 | 0.000719 | 0.205 |
| wt P31 7 | 1170000 | 5 | 4583 | 0.000459 | 0.21 |
| wt P31 8 | 512000 | 2 | 1516 | 0.000414 | 0.179 |
| wt P31 9 | 638000 | 4 | 2446 | 0.000847 | 0.226 |
| **average** | **1200333.33** | **5.44** | **3076.22** | **0.000453** | **0.154** |
|  |  |  |  |  |  |
| rd10 P31 1 | 505000 | 443 | 12446 | 0.0856 | 1.21 |
| rd10 P31 2 | 255288 | 145 | 2014 | 0.0452 | 0.656 |
| rd10 P31 3 | 532000 | 504 | 6351 | 0.0897 | 0.944 |
| rd10 P31 4 | 292137 | 275 | 3679 | 0.0843 | 0.981 |
| rd10 P31 5 | 618000 | 262 | 3911 | 0.0393 | 0.462 |
| rd10 P31 6 | 590000 | 141 | 3470 | 0.0272 | 0.436 |
| **average** | **465404.16** | **295** | **5311.83** | **0.0618** | **0.781** |
|  |  |  |  |  |  |
| wt P42 1 | 818000 | 5 | 1437 | 0.000557 | 0.101 |
| wt P42 2 | 1090000 | 3 | 2356 | 0.000202 | 0.129 |
| wt P42 3 | 1220000 | 3 | 3261 | 0.00037 | 0.161 |
| wt P42 4 | 1060000 | 2 | 1900 | 0.000103 | 0.103 |
| wt P42 5 | 1090000 | 6 | 6148 | 0.000388 | 0.192 |
| wt P42 6 | 915000 | 2 | 4993 | 0.000231 | 0.241 |
| wt P42 7 | 843000 | 6 | 3679 | 0.000639 | 0.241 |
| **average** | **1005142.86** | **3.85** | **3396.28** | **0.000355** | **0.166** |
|  |  |  |  |  |  |
| rd10 P42 1 | 340834 | 125 | 4222 | 0.0358 | 0.733 |
| rd10 P42 2 | 260542 | 121 | 5227 | 0.0516 | 0.996 |
| rd10 P42 3 | 168766 | 75 | 2092 | 0.054 | 0.975 |
| rd10 P42 4 | 265520 | 128 | 3272 | 0.0494 | 0.96 |
| rd10 P42 5 | 57553 | 42 | 880 | 0.0784 | 1.32 |
| rd10 P42 6 | 143457 | 67 | 1523 | 0.0468 | 0.875 |
| rd10 P42 7 | 285938 | 125 | 3499 | 0.0433 | 0.902 |
| rd10 P42 8 | 701000 | 146 | 5305 | 0.0214 | 0.485 |
| rd10 P42 9 | 613000 | 91 | 3439 | 0.0148 | 0.382 |
| **average** | **315178.88** | **102.22** | **3273.22** | **0.0439** | **0.847** |
|  |  |  |  |  |  |
