# Supplementary material for: Circulating inflammatory monocytes oppose microglia and contribute to cone cell death in retinitis pigmentosa
Source: PNAS Nexus. 2022 Mar 2;1(1):pgac003. doi: 10.1093/pnasnexus/pgac003 (PMC9075747; doi:10.1093/pnasnexus/pgac003)
Supplement: pgac003_Supplemental_Files [file pgac003_supplemental_files.zip › PNASNEXUS-PNASNEXUS-2021-00163-s03.docx]

**Table S2. Chemokines/receptors related genes that are significantly differentially expressed in rd10 compared to WT are shown.**

|  | **Log2 fold change** | **P-value** |
| --- | --- | --- |
| **Csf1** | 1.75 | 8.16E-06 |
| **Cx3cl1** | 1.3 | 8.18E-06 |
| **Cx3cr1** | 2.93 | 8.11E-06 |
| **Ifnar1** | 0.228 | 0.0115 |
| **Ifnar2** | 1.17 | 2.9E-05 |
| **Il1r1** | 1.63 | 3.59E-05 |
| **Il1rap** | 0.937 | 0.000345 |
| **Il2rg** | 0.676 | 0.0112 |
| **Ltbr** | 1.81 | 8.44E-06 |
| **Ngf** | 0.811 | 0.01 |
| **Ngfr** | 1.16 | 0.000139 |
| **Osmr** | 2.34 | 1.02E-07 |
| **Tgfb1** | 1.58 | 0.000614 |
| **Tgfbr1** | 0.431 | 0.000202 |
| **Tnfrsf1a** | 2.72 | 5.15E-07 |
| **Tnfsf12** | -1.83 | 1.93E-05 |
