# Supplementary material for: Circulating inflammatory monocytes oppose microglia and contribute to cone cell death in retinitis pigmentosa
Source: PNAS Nexus. 2022 Mar 2;1(1):pgac003. doi: 10.1093/pnasnexus/pgac003 (PMC9075747; doi:10.1093/pnasnexus/pgac003)
Supplement: pgac003_Supplemental_Files [file pgac003_supplemental_files.zip › PNASNEXUS-PNASNEXUS-2021-00163-s04.docx]

**Table S3. The absolute numbers of microglia and mφ as well as the total numbers of live cells in the retinas (rd10 P31; pbs vs FITC-NPs vs PVS-NPs).**

|  | live cells | mφ count | microglia count | mφ/live cells % | microglia/ live cells % |
| --- | --- | --- | --- | --- | --- |
| pbs 1 | 239536 | 247 | 2423 | 0.103 | 1.012 |
| pbs 2 | 257717 | 269 | 2575 | 0.104 | 0.999 |
| pbs 3 | 181909 | 155 | 1589 | 0.085 | 0.874 |
| pbs 4 | 175096 | 154 | 1726 | 0.088 | 0.986 |
| pbs 5 | 36425 | 69 | 549 | 0.189 | 1.507 |
| pbs 6 | 83639 | 186 | 1871 | 0.222 | 2.237 |
| pbs 7 | 69530 | 325 | 1744 | 0.467 | 2.508 |
| pbs 8 | 73688 | 241 | 1599 | 0.327 | 2.170 |
| pbs 9 | 75088 | 246 | 1678 | 0.328 | 2.235 |
| pbs 10 | 71932 | 164 | 1441 | 0.228 | 2.003 |
| pbs 11 | 106162 | 190 | 1504 | 0.179 | 1.417 |
| pbs 12 | 81791 | 177 | 1409 | 0.216 | 1.723 |
| pbs 13 | 245548 | 375 | 2582 | 0.153 | 1.052 |
| pbs 14 | 200873 | 380 | 2192 | 0.189 | 1.091 |
| **average** | **135638** | **227** | **1777** | **0.206** | **1.558** |
|  |  |  |  |  |  |
| FITC 1 | 169966 | 190 | 1862 | 0.112 | 1.096 |
| FITC 2 | 186719 | 181 | 1917 | 0.097 | 1.027 |
| FITC 3 | 172916 | 203 | 1632 | 0.117 | 0.944 |
| FITC 4 | 201245 | 272 | 2036 | 0.135 | 1.012 |
| FITC 5 | 117420 | 340 | 2218 | 0.290 | 1.889 |
| FITC 6 | 132714 | 532 | 3203 | 0.401 | 2.413 |
| FITC 7 | 144858 | 255 | 2618 | 0.176 | 1.807 |
| FITC 8 | 115933 | 320 | 2526 | 0.276 | 2.179 |
| FITC 9 | 121257 | 462 | 3163 | 0.381 | 2.609 |
| FITC 10 | 142422 | 341 | 3337 | 0.239 | 2.343 |
| FITC 11 | 127749 | 113 | 1191 | 0.088 | 0.932 |
| FITC 12 | 148476 | 91 | 1377 | 0.061 | 0.927 |
| **average** | **148473** | **275** | **2257** | **0.198** | **1.598** |
|  |  |  |  |  |  |
| PVS 1 | 155709 | 151 | 1724 | 0.097 | 1.107 |
| PVS 2 | 179683 | 138 | 1985 | 0.077 | 1.105 |
| PVS 3 | 168312 | 189 | 1623 | 0.112 | 0.964 |
| PVS 4 | 190832 | 254 | 1922 | 0.133 | 1.007 |
| PVS 5 | 195773 | 257 | 2786 | 0.131 | 1.423 |
| PVS 6 | 132636 | 170 | 2107 | 0.128 | 1.589 |
| PVS 7 | 204790 | 124 | 1412 | 0.061 | 0.689 |
| PVS 8 | 374872 | 276 | 3049 | 0.074 | 0.813 |
| PVS 9 | 123722 | 176 | 2109 | 0.142 | 1.705 |
| PVS 10 | 240815 | 288 | 3930 | 0.120 | 1.632 |
| PVS 11 | 117835 | 184 | 2114 | 0.156 | 1.794 |
| PVS 12 | 195475 | 234 | 3610 | 0.120 | 1.847 |
| PVS 13 | 201142 | 210 | 1520 | 0.104 | 0.756 |
| PVS 14 | 375254 | 236 | 2918 | 0.063 | 0.778 |
| **average** | **204061** | **206** | **2344** | **0.108** | **1.229** |
