# Supplementary figures and images for: Circulating inflammatory monocytes oppose microglia and contribute to cone cell death in retinitis pigmentosa
Source: PNAS Nexus. 2022 Mar 2;1(1):pgac003. doi: 10.1093/pnasnexus/pgac003 (PMC9075747; doi:10.1093/pnasnexus/pgac003)

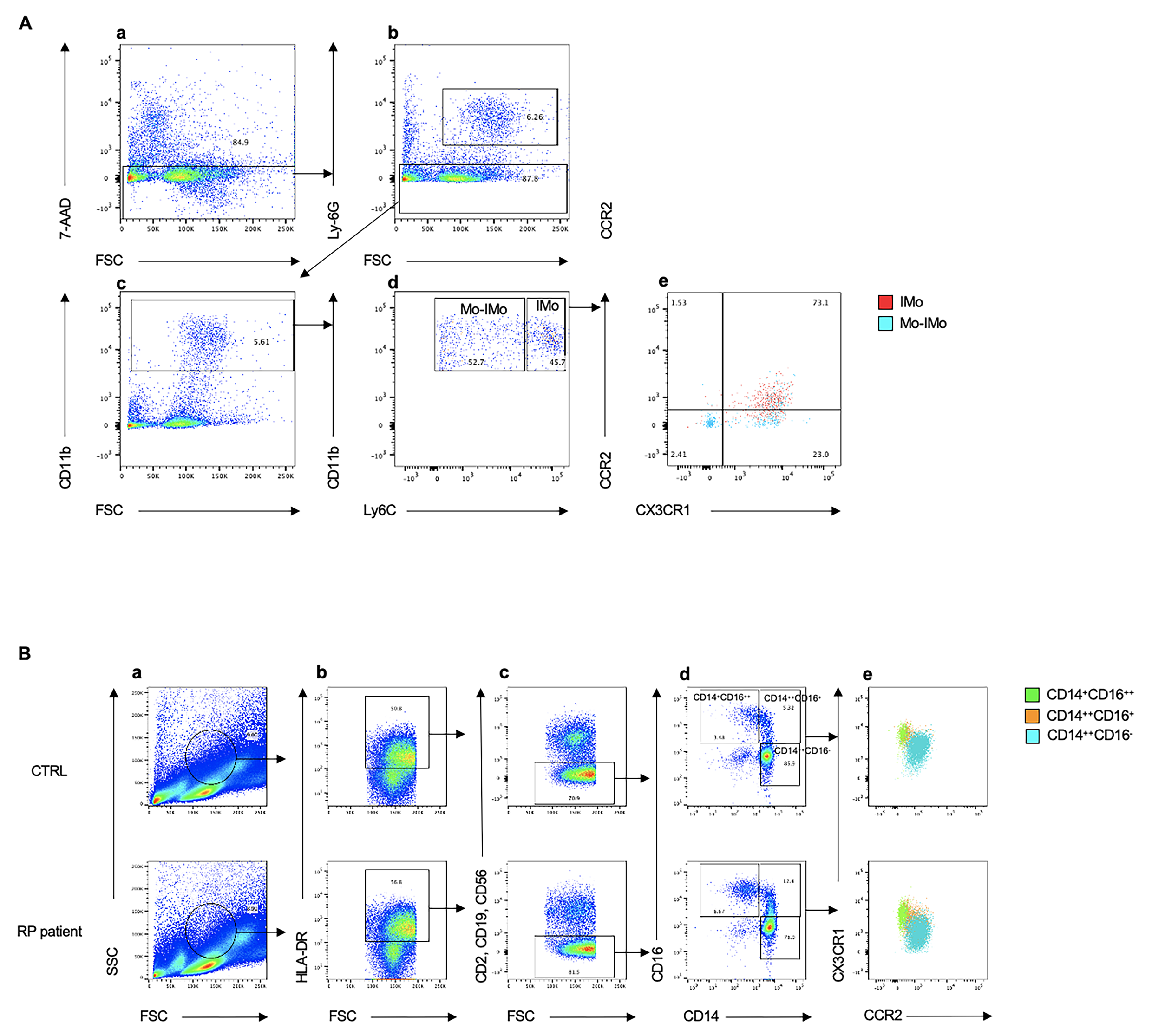

Supplement: pgac003_Supplemental_Files [file pgac003_supplemental_files.zip › PNASNEXUS-PNASNEXUS-2021-00163-s05.tif]

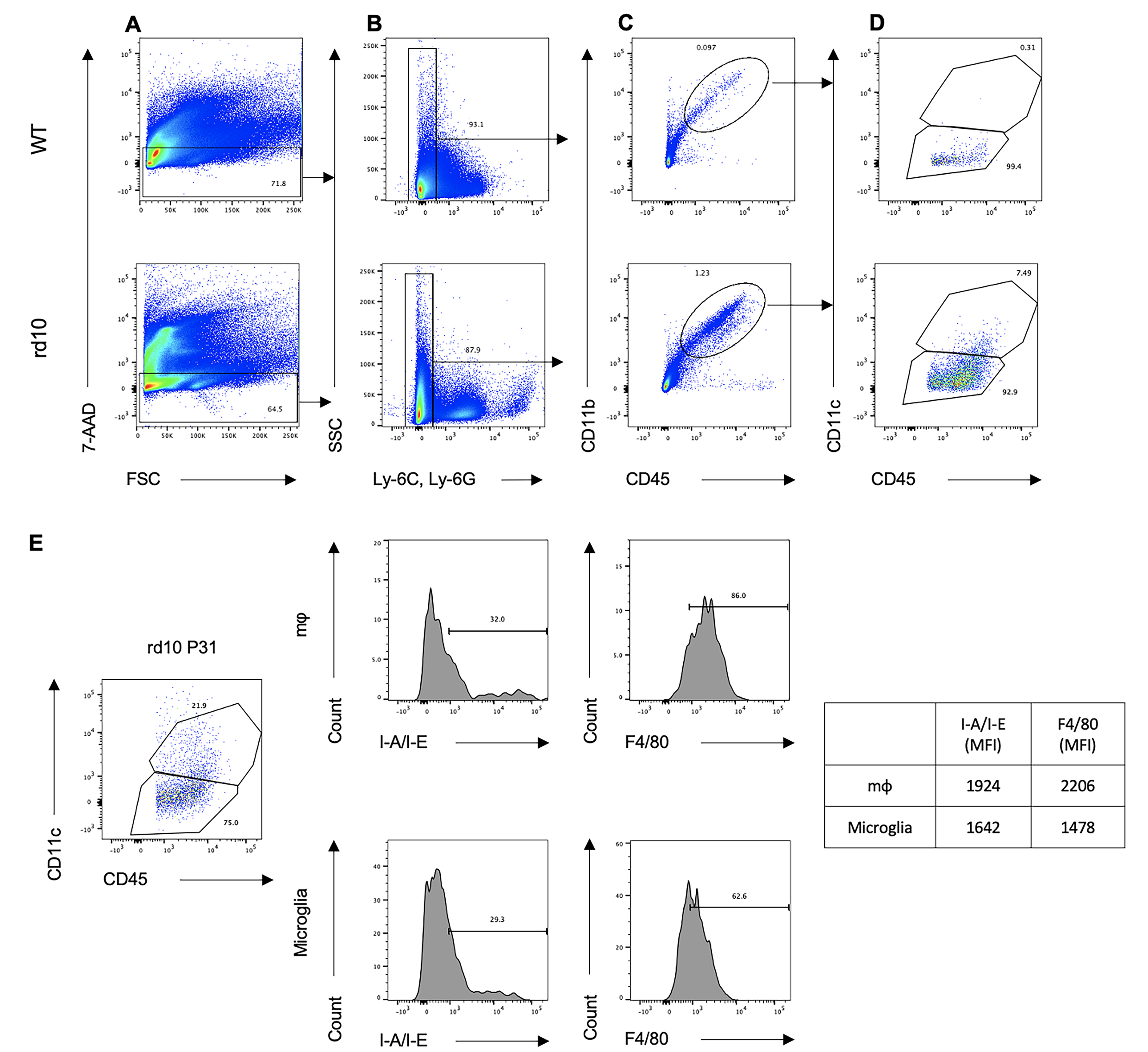

Supplement: pgac003_Supplemental_Files [file pgac003_supplemental_files.zip › PNASNEXUS-PNASNEXUS-2021-00163-s06.tif]

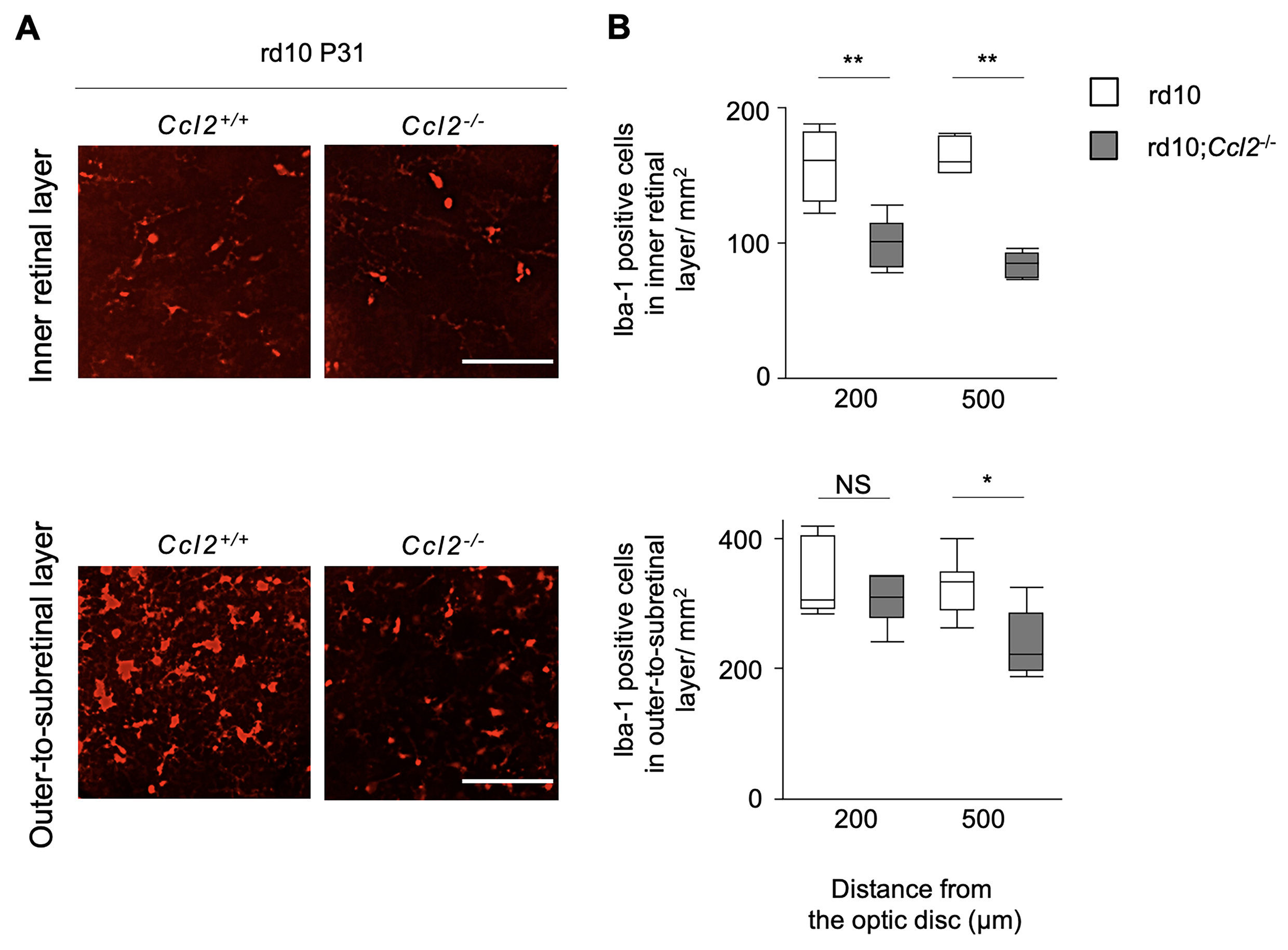

Supplement: pgac003_Supplemental_Files [file pgac003_supplemental_files.zip › PNASNEXUS-PNASNEXUS-2021-00163-s07.tif]

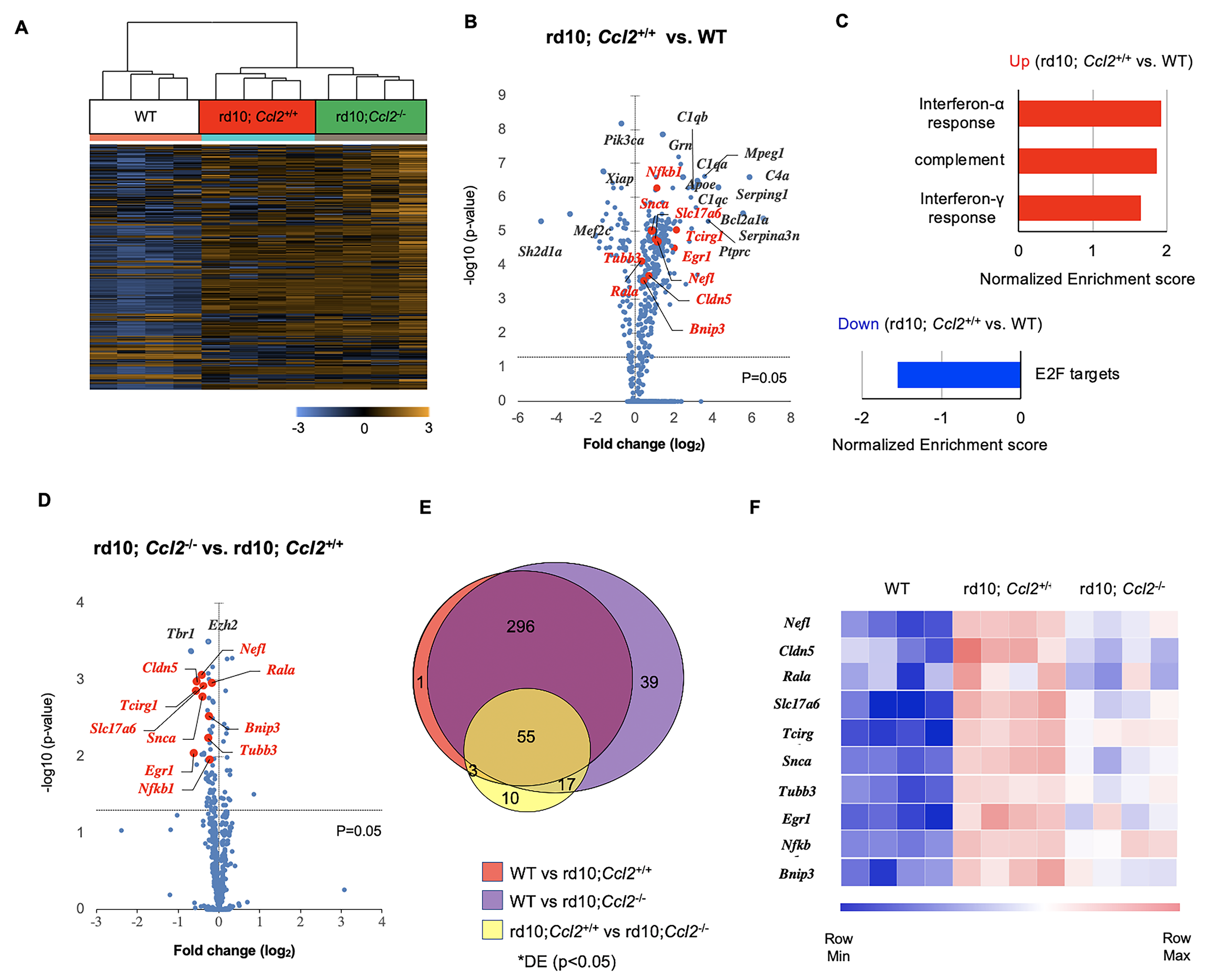

Supplement: pgac003_Supplemental_Files [file pgac003_supplemental_files.zip › PNASNEXUS-PNASNEXUS-2021-00163-s08.tif]

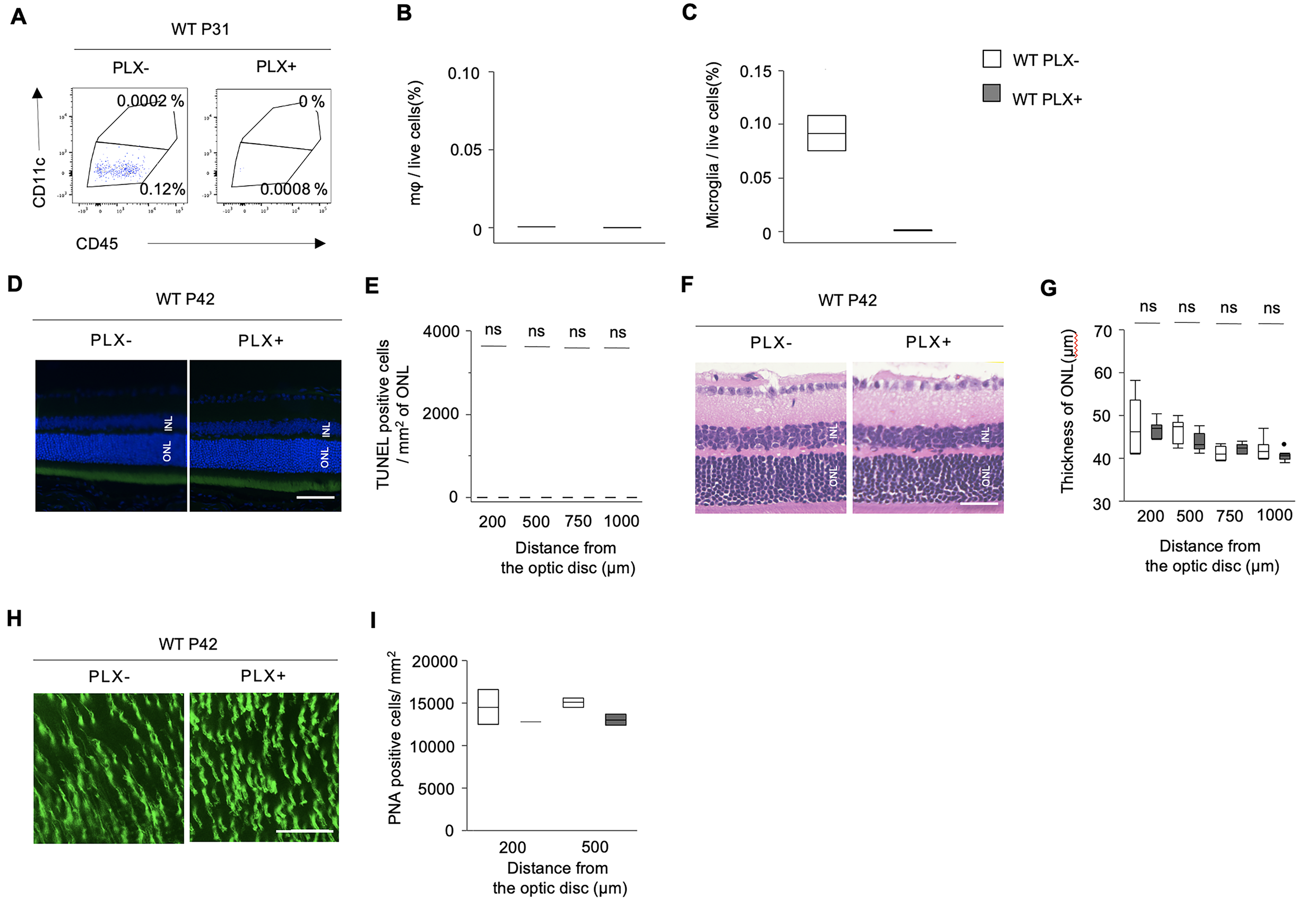

Supplement: pgac003_Supplemental_Files [file pgac003_supplemental_files.zip › PNASNEXUS-PNASNEXUS-2021-00163-s09.tif]

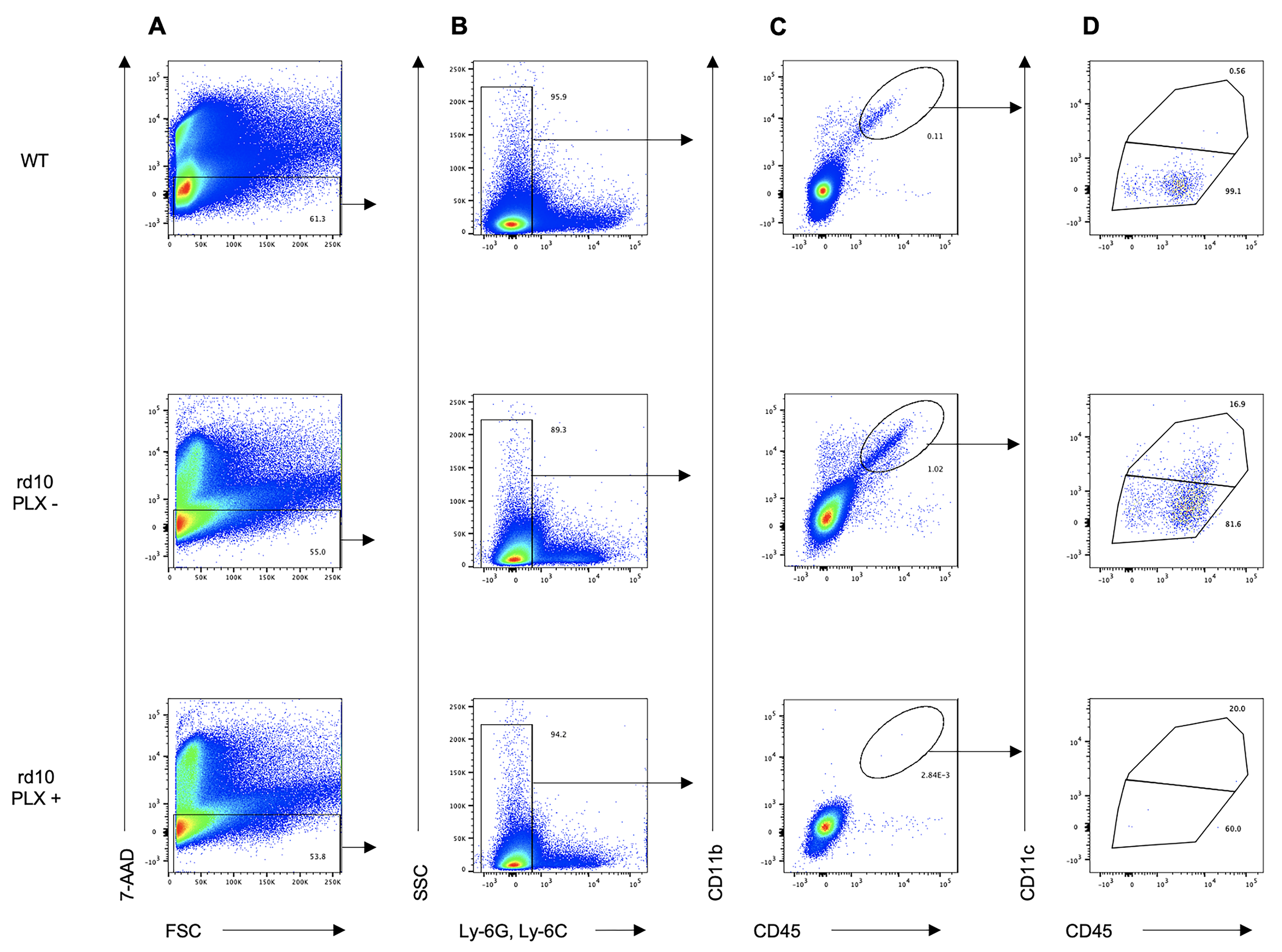

Supplement: pgac003_Supplemental_Files [file pgac003_supplemental_files.zip › PNASNEXUS-PNASNEXUS-2021-00163-s10.tif]

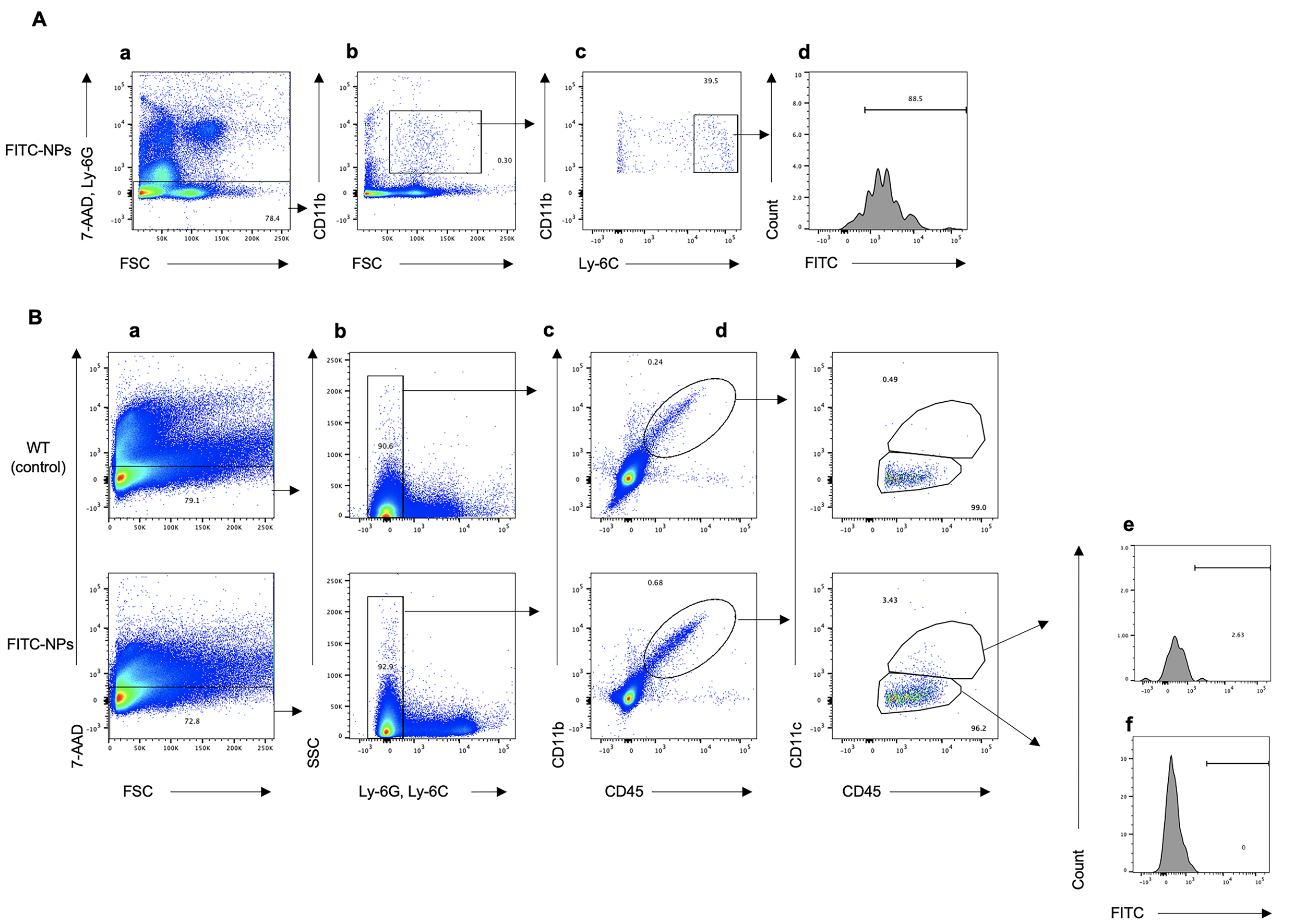

Supplement: pgac003_Supplemental_Files [file pgac003_supplemental_files.zip › PNASNEXUS-PNASNEXUS-2021-00163-s11.tif]

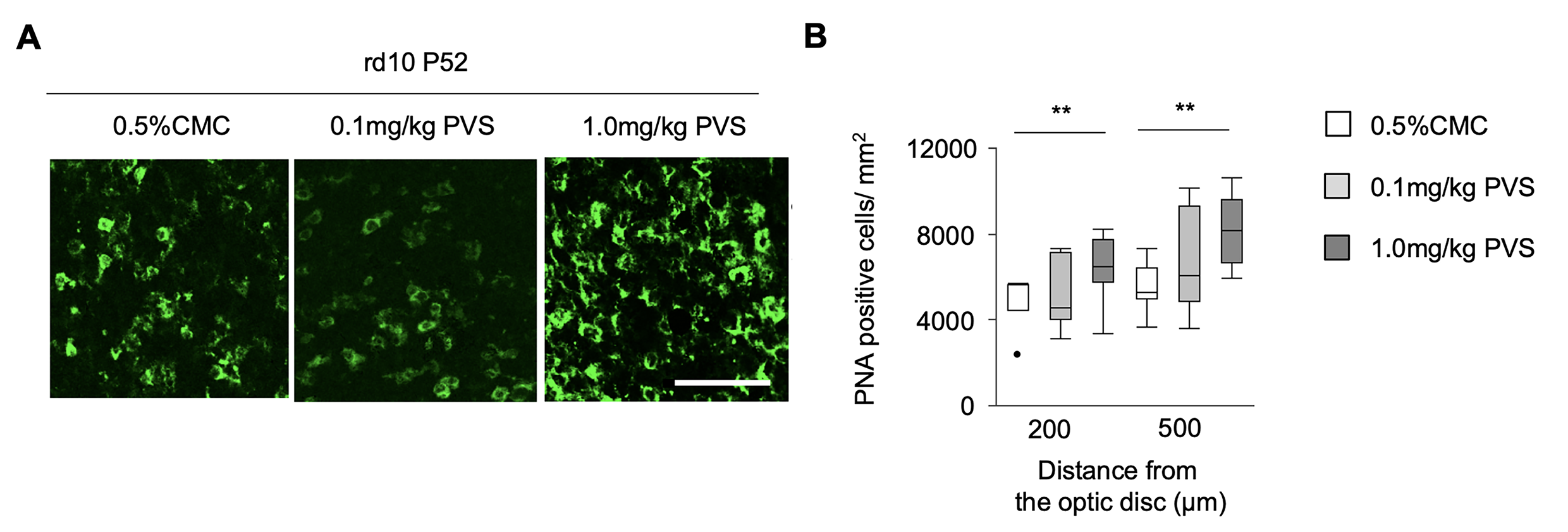

Supplement: pgac003_Supplemental_Files [file pgac003_supplemental_files.zip › PNASNEXUS-PNASNEXUS-2021-00163-s12.tif]
